# Supplementary material for: Ultra-stretchable and biodegradable elastomers for soft, transient electronics
Source: Nat Commun. 2023 Apr 20;14:2263. doi: 10.1038/s41467-023-38040-4 (PMC10119106; doi:10.1038/s41467-023-38040-4)
Supplement: Supplementary file 1 — Supplementary Information [file 41467_2023_38040_MOESM1_ESM.pdf]

Supplementary Information

Ultra-Stretchable and Biodegradable Elastomers for Soft, Transient Electronics

Won Bae Han[1], Gwan-Jin Ko[1], Kang-Gon Lee[2], Donghak Kim[3], Joong Hoon Lee[1], Seung Min Yang[1,4], Dong-Je Kim[1], Jeong-Woong Shin[1], Tae-Min Jang[1], Sungkeun Han[1], Honglei Zhou[5], Heeseok Kang[1], Jun Hyeon Lim[1], Kaveti Rajaram[1], Huanyu Cheng[5], Yong-Doo Park[2], Soo Hyun Kim[3], and Suk-Won Hwang [1,3,6,]*

[1] KU-KIST Graduate School of Converging Science and Technology, Korea University, 145 Anam-ro, Seongbuk-gu, Seoul, 02841, Republic of Korea

[2] Department of Biomedical Sciences, College of Medicine, Korea University, 145 Anam-ro, Seongbuk-gu, Seoul, 02841, Republic of Korea

[3] Center for Biomaterials, Biomedical Research Institute, Korea Institute of Science and Technology (KIST), 5 Hwarang-ro 14-gil, Seongbuk-gu, Seoul, 02792, Republic of Korea

[4] Hanwha Systems Co., Ltd., 188, Pangyoyeok-ro, Bundang-gu, Seongnam-si, Gyeonggi-do, 13524, Republic of Korea

[5] Department of Engineering Science and Mechanics, The Pennsylvania State University, University Park, PA, 16802, USA

[6] Department of Integrative Energy Engineering, Korea University, 145 Anam-ro, Seongbuk-gu, Seoul, 02841, Republic of Korea

[†]These authors equally contributed to this work

*Correspondence to: dupong76@korea.ac.kr

Supplementary Notes

Supplementary Note 1. Preparation of poly(L-lactide-co-ε-caprolactone) (PLCL) film

A PLCL solution was prepared by dissolving the PLCL in dimethylformamide (DMF) at a concentration of 15 w/v%. An appropriate amount of the PLCL solution was drop-casted onto a polydimethylsiloxane (PDMS, Sylgard 184, Dow corning, USA) mold bonded to a glass substrate, followed by drying in ambient conditions for >12 h and vacuum for 3 h to obtain a clear and transparent PLCL film. For visualization in stretching experiments, 0.1 mol% of the fluorescent dye, Nile red (Sigma-Aldrich, USA), was added to the PLCL solution.

Supplementary Note 2. Biodegradation test

PLCL films (100 μm-thick; length: 15 mm; width: 15 mm) were freshly made right before tests. The degradation rates for enzymatic and chemical hydrolysis were monitored by comparing the weight of PLCL before and after degradation. The percent weight ratio was calculated with the formula: % weight ratio = W_2 / W_1 × 100, where W_1 and W_2 are the pre- and post-degradation weights of PLCL films, respectively.

Enzymatic degradation. Saprotrophic fungus, *Aspergillus flavus* (KCTC16682), placed on the center of a medium, malt extract agar (KCTC media no. 36), in a petri dish was purchased from the Korean Collection for Type Cultures and used as received. Preweighed PLCL films (140k) were placed around the fungus, and the petri dish was then incubated in an incubator (Mini microbiology and hematology oven, LABNET international, USA) with a humidity and temperature of 50 % and 37 °C for 30 days. Over time, each film was taken out, thoroughly rinsed with deionized (DI) water, dried in a vacuum oven at 50 °C for 24 h, and weighed to calculate the degradation rate of PLCL.

Dissolution test. Preweighted PLCL films (60k, 140k, and 200k) were immersed into a bath of various solutions, such as DI water, 0.1 M of phosphate-buffered-saline (PBS, pH 3, 7, or 11, Sigma-Aldrich, USA), and artificial cerebrospinal fluid (ACSF, Ecocyte Bioscience), at room temperature (RT) and different temperatures of 37 °C and 50 °C for 30 weeks. Each film was removed every 2 weeks, rinsed with DI water, dried in a vacuum oven at 50 °C for 24 h, and weighted to calculate the percent weight loss.

Supplementary Note 3. Characterization of mechanical/physical properties

PLCL films (100 μm-thick) were freshly made right before tests and all the tests were performed in ambient conditions if not mentioned otherwise.

Uniaxial and biaxial stretching. PLCL films (60k; length: 20 mm; width: 10mm) were stretched linearly, or polygonally along metal poles in heart and star shapes. For biaxial stretching, a PLCL film was bonded to an aperture (diameter: 10 mm) formed in a 3D printed-poly(lactic acid) (PLA) chamber, and a positive pneumatic pressure was supplied through the aperture to inflate the PLCL film until material failure. The areal strain was calculated from the diameter of the film before and after the inflation.

Mechanical properties. Mechanical tensile tests were conducted by a universal mechanical testing system (Instron 5900 series, USA) with a constant stretching rate of 6 mm/min on free-standing PLCL films (60k, 140k, and 200k; length: 20 mm; width: 10 mm). The Young's moduli (E) and the toughness were defined as the slopes of stress-strain curves within the elastic range and the area under the recorded stress-strain curves with strain from 0 to strain at break (ε_b), respectively. For measuring fracture energy, a notch was introduced on the one side of the PLCL film at a length of 5 mm. The fracture energy was calculated with the

formula: fracture energy, $G_n = 6 \times W \times c / \varepsilon_n^{1/2}$, where c and ε_n are notch length and strain at break of the notched sample, respectively, and W is the toughness of the original sample for strain from 0 to ε_n. To study the effect of biodegradation and storage time on the mechanical properties of PLCL, PLCL films (140k; length: 20 mm; width: 10 mm) were prepared by dissolving in PBS (pH 7) at 37 °C until reached to degradation ratios of 15 %, 30 %, and 40 % and by storing in ambient conditions (~20 °C; RH ~40 %) for 1 and 6 months, respectively. And the stress-strain curve of the PLCL film was measured and compared with one of the original PLCL film to calculate the normalized values.

Water uptake. PLCL films (60k, 140k, and 200k; length and width: 15 mm) were immersed in PBS (pH 7) at 37 °C for 5 weeks. Each sample was removed every 1 week, swabbed using filter paper, weighted to determine M_s, and then dried in a vacuum oven at 50 °C for 24 h to determine M_d. Water uptake was quantitatively described by swelling percentage, defined as $(M_s - M_d) / M_d$.

Optical properties. Optical transmittances of PLCL (140k and 200k) and PDMS was scanned with a wavelength from 250 to 850 nm by UV-visible spectroscopy (8453E, Agilent Technologies, USA).

Dielectric constant. The parallel plate method (ASTM D150) was used to measure the dielectric constant of PLCL as follows. PLCL film was sandwiched between two electrodes to form a capacitor, and the capacitance was measured with a wavelength range from 4 Hz to 200 kHz by an LCR meter (IM3533-01, HOIKI E.E Corporation, Japan). Dielectric constant, $\varepsilon_{r'}$, was defined as $(t \times C_p) / (A \times \varepsilon_0)$, where t is the thickness of the PLCL film, C_p is the measured capacitance, A is the area of the electrode, and ε_0 is the permittivity of vacuum.

Adhesion energy to various materials. 180-degree peeling tests (ASTM F2256) were used to evaluate the adhesion between bonded pieces of PLCLs (140k and 200k; length: 20 mm; width: 10 mm) and between PLCLs and other materials, including PDMS, silicon (Si), silicon dioxide (SiO_2), and magnesium (Mg), formed on glass substrates. In all cases, a layer of PLA (50 µm-thick) was used as stiff backing for PLCL films to eliminate the energy dissipation from an elastic deformation. The Instron machine was used to apply unidirectional tension with a constant loading rate of 60 mm/min while recording the force-strain curves. The adhesion energy was calculated by twice the plateau value of the ratio of the tensile force and the width of the film.

Thermal properties. Differential scanning calorimetry (DSC) thermograms were obtained using a DSC 2010 (TA Instruments, USA) at a heating rate of 10 °C/min from -70 to 200 °C. Values of glass transition temperatures (T_g) were taken from the midpoints of the transition zones. The thermal stability was analyzed by performing the thermogravimetric analysis (TGA) using an SDT Q600 (TA Instruments, USA) at a heating rate of 5 °C/min from 25 to 500 °C in an N_2 atmosphere. The coefficients of thermal expansion were measured based on ASTM E831 by a TMA Q400 (TA Instruments, USA) with a load of 0.05 N at a heating rate of 5 °C/min from 25 to 200 °C in an N_2 atmosphere.

Solvent compatibility. Preweighted films (500 µm-thick; length and width: 5 mm) of PLCL (140k and 200k), poly(lactide-co-glycolic acid) (PLGA), and PDMS were swollen in various solvents, including ethanol, isopropanol (IPA), acetone, nitric acid (HNO_3), and hydrofluoric acid (HF) for 5 mins in ambient conditions, swabbed using filter paper, and weighted to determine the volumes of solvents swollen into the films. Solvent compatibility was described

by the degree of swelling, defined as the ratio of the swollen volume of solvent and the weight of the original film.

Supplementary Note 4. Fabrication of a flexible 8×16 RGB light-emitting diode (LED) matrix array on PLCL

A temporary substrate was prepared by spin coating bilayer of poly(methylmethacrylate) (PMMA, MicroChem, USA; ~100 nm thick) and polyimide (PI, Sigma-Aldrich, USA; ~1.2 μm thick) on a Si wafer (p-type, Silicon Technology Co., Japan). The bottom electrodes (Cr 5 nm/Au 100 nm) were deposited and patterned on the substrate by e-beam evaporation and photolithography. After spin-coating a layer of diluted PI (D-PI, ~400 nm thick) as a flexible insulator, the top electrodes (Cr 5 nm/Au 100nm) were formed. Another insulating layer of D-PI was spin-coated to encapsulate the top electrodes, and dry reactive ion etching (RIE, JVAC, South Korea) defined the mesh pattern of electrodes and created openings for electrical contact pads of LEDs and external interfaces. Removal of the PMMA layer by immersing the substrate in acetone enabled releasing and subsequent transfer-printing of the entire device on a film of PLCL (140k, ~500 μm thick). LEDs (surface mount device, 0402 package) with red, green, and blue colors were placed on the openings for LEDs and electrically connected to the bottom and top electrodes using silver conductive epoxy. After bonding an ACF cable to the exposed electrodes for interfacing with an Arduino microcontroller, a prefabricated film of PLCL (140k, ~100 μm-thick) was covered on top of the device for encapsulation.

Supplementary Note 5. Fabrication of a biodegradable, stretchable complemenatry metal-oxide-semiconductor (CMOS) array on PLCL

Device fabrication began with three different doping processes on p-type silicon-on-insulator (SOI (top Si ~300 nm), SOITECH, France) wafers. Phosphorus doping at 950 °C with a spin-

on dopant (SOD, Filmtronics, USA) defined heavily doped n+ regions for the source and drain contacts for n-type transistors, and lightly doped n-type regions for the n-wells (n-) were formed at 650 °C. Highly doped p-type areas for source and drain electrodes of p-type transistors were formed at 1050 °C with boron doping. Wet etching of a box oxide using hydrofluoric acid (HF, Transene Company Inc., USA) released the top silicon nanomembranes (Si NMs) from the SOI, enabling transfer printing onto a PMMA/D-PI spin coated on a temporary carrier substrate. Active areas of the doped Si NMs for transistors were isolated by RIE with sulfur hexafluoride gas (SF_6). A thin layer of SiO_2 for the gate dielectric was deposited by plasma-enhanced chemical vapor deposition (PECVD, Plasmalab 800 plus, Oxford, UK), and the contact pad opening process for the source and drain electrodes of the devices was performed by wet etching using a buffered oxide etchant (BOE, Transene Company Inc., USA). A layer of Mg (~300 nm thick) deposited by e-beam evaporator (VER5004, South Korea) was used for the source, drain, gate electrodes, as well as interconnects. Another thin dielectric layer of SiO_2 encapsulated the device, except for source, drain, and gate contact pads, followed by deposition of devices contact electrodes with Mg using e-beam evaporation. A layer of D-PI was spin-coated on top of the patterned structure stack. A mesh-type geometric pattern was formed through dry etching with O_2 RIE, and the array of CMOS can be easily lifted off the temporary substrate by immersing it in acetone. After removing the bottom D-PI layer, the device was transfer printed onto a film of PLCL (140k, ~200 μm-thick), followed by removal of the top D-PI layer.

Supplementary Note 6. Direct fabrication of a microheater array on PLCL by microlithography

A PLCL substrate (200k, ~200 μm-thick) was formed through drop casting of PLCL solution on the hexamethyldisilazane (HMDS, Sigma Aldrich, USA) treated surface of a carrier Si wafer.

Coating and patterning of photoresist (PR, APOL-LO 3207, KemLab, USA) by photolithography defined a device pattern on the PLCL substrate. A 300 nm-thick of Mg was deposited on the patterned substrate by sputtering (DC sputtering system, KVS-2004, South Korea), and the lift-off process was performed by immersion in ethanol. After rinsing with ethanol and blowing with N_2, the device was physically peeled off the carrier wafer to complete the fabrication.

Supplementary Note 7. Fabrication of PLCL/PP-based electronics

Fabrication of partially biodegradable, stretchable electronic circuits. PDMS masks (200 μm-thick) with patterns of electronic circuits (feature size: ~500 μm) were fabricated by laser-cutting (Epilog Legend Helix, Epilog laser, USA). 1 w/v% of PLCL/PP ink (0.6:0.4:1) was drop-casted on PDMS/glass substrate through the PDMS mask and partially dried at 80 °C for 3 h. These steps were repeated three times. After gently removing the mask, the 15 w/v% PLCL solution was further drop-casted as a supporting layer and fully dried at 80 °C for 12 h. A LED was electrically connected to the PLCL/PP pattern using a silver conductive paste, and a piece of rigid PLCL film (LA:CL, 8:2; Mn, 200k; 100 μm-thick; length and width: 3 mm) was used as a strain-free rigid island for the LED.

Electrocardiogram (ECG) and electromyogram (EMG) measurements. Similar to the process described above, sensing probes for ECG and EMG were fabricated based on PLCL/PP (detailed structure appears in Supplementary Fig. S13). All signals were simultaneously measured by placing both PLCL/PP and commercial Ag/AgCl probes on similar locations. According to the lead I of standard limb leads, the ECG was acquired for 30 s by attaching source and reference probes on the inner left and right arms, respectively, and a ground probe on the inner left foot firmly with a transparent film dressing (Tegaderm, 3M, USA). For EMG

measurements, source and reference probes were placed at a longitudinal spacing of 10 mm in the center of the flexor carpi radialis on the inner left arm, and a ground probe was attached on the inner left wrist. The EMG was acquired by repeatedly clenching and opening the left fist four times. All signals were amplified by a factor of 1000 using an amplifier (Model 1700 differential AC amplifier, A-M Systems, USA), processed with a bandpass filter (0.5 - 100 Hz) and notch filter to reduce power line interference, and obtained at a sampling frequency of 1 kHz by a NI DAQ (National instrument, USA). The signals were further processed with the third-order Butterworth bandpass filter (0.1 - 40 Hz) using Matlab (MathWorks, USA).

Supplementary Note 8. Fabrication of PLCL/Mo-based electronics

Voice and pulse recordings. The sensors were fabricated by trimming the prepared PLCL/Mo (9:1) into a strip (length: 25 mm; width: 3 mm) and connecting ACF cables to the two ends of the strip for external interfaces. The sensors were attached to the human's neck and wrist firmly with the Tegaderm for voice and pulse recordings, respectively, and the changes in resistance were measured by a source meter (Keithley 2636b, Tektronix, USA) at a sampling frequency of 1 kHz. For voice recording, three speakers were asked to repeat three different words ("Hello", "Korea", "Happy") more than ten times, while pulse signals were obtained before and after strenuous running for 30 min.

Fabrication of pressure sensor array. Methods similar to those used to manufacture LED arrays were applied to fabricate top and bottom crossed arrays of electrodes containing 6 × 6 via holes with exposed electrodes, which were picked up by a PDMS stamp. The PLCL/Mo ink (9:1) was drop-casted into the holes and dried at 80 °C for 12 h, followed by transfer-printing the device onto a prefabricated PLCL spacer. ACF cables were connected to the device for interfacing with the source meter, and changes in resistance were monitored in real-

time by placing several objects on the device, such as cotton balls, a coffee bean, and a ladybird toy.

Supplementary Note 9. Transient, smart soft actuator

Pressure control and temperature/pressure sensing system. To operate the smart gripper according to customized operational principles and algorithm flowchart as described in Supplementary Fig. S23, we established a pressure control system (Supplementary Fig. S19). The system included a compressor, a pressure regulator, solenoid valves, a relay module, and a microcontroller unit (MCU). The MCU read temperature and pressure levels measured by the sensors integrated on the gripper and operated the solenoid valves. Solenoid valves switched on/off pneumatic pressure generated by the compressor.

Characterization of bending actuation. Positive pressure was delivered to the gripper by a compressor (Beetle bug, Denkou Syounendan, Japan) while simultaneously recording the bending motion and measuring the applied pressures by a pressure gauge (PGW-500, Elitech, USA). Cyclic actuation stability was examined with the established pressure control system.

Characterization of temperature/pressure sensors. The temperature sensor was placed on a hotplate and gradually heated while monitoring the change in resistance by the source meter. The pressure sensor was characterized by monitoring the change in resistance for PDMS blocks with specific dimensions/weights targeting 0.05, 0.1, 0.2, 0.5, and 1.0 kPa. The sensing stabilities were evaluated by actuating the gripper at a bending angle of ~90 ° during the measurements.

Finite element simulation. Full three-dimensional (3D) finite element analysis (FEA) was performed to analyze the bending behavior of the PLCL (140k)-based actuator (dimension shown in Fig. S19) in the commercial software ABAQUS (ABAQUS 2017). A linear elastic model was used to characterize the deformation behaviors of the PLCL (140k) with Young's modulus of ~3 MPa. The 8-node linear brick elements (ABAQUS element type C3D8) were chosen to mesh the model, giving a total number of 45,781 elements with a minimum element size of 0.4 mm. After setting normal hard contact property in the self-contact for the entire PLCL (140k) actuator, pressure load was uniformly applied on the inner surfaces of the pneumatic chambers. The increasing applied pressure leads to the increased bending angle, which further results in the increased average strain in the PLCL (140k) actuator (Figs. 4b and 4c).

Fabrication of a soft gripper based on PLCL elastomers with two different moduli. A low-modulus semi-open actuator (60k; width, 5 mm; height, 5 mm; length, 40 mm; thickness, 500 μm) and a high-modulus flat film (200k; width, 5 mm; length, 40 mm; thickness, 500 μm) were prepared by molding, bonded together, and assembled with a 3D-printed PLA head connecting to a pneumatic source. When positive pressure was applied, the 60k-based layer expanded more than the 200k-based layer due to the lower mechanical moduli, which caused the actuator to bend.

Fabrication of artificial muscle. The artificial muscle was fabricated by bonding a PLCL membrane (200k; ~500 μm-thick) to the hole (diameter, 20 mm) of a 3D printed PLA head using a small amount of the PLCL solution as adhesive. Applying negative/positive pressure activated the artificial muscle to raise or lower the weights of 50 g and 100 g.

Supplementary Note 10. Soft, transient, suture-free cardiac jacket

Structural design optimization of open-mesh body and stingray barb-inspired straps. For open-mesh body, PLCL films (140k; ~50 μm-thick) were cut into a mesh and a serpentine mesh structures as shown in Fig. 5c. A pristine PLCL film and the cut mesh and serpentine mesh were tested by the universal mechanical testing system with a constant stretching rate of 6 mm/min. For stingray barb-inspired straps, male parts with different lengths and angles of barbs and corresponding female parts were prepared by laser-cutting a PLCL film (140k; ~50 μm-thick). The fastening/releasing forces of each sample were measured by inserting/removing a male part into/from a female part, respectively, using the tensile machine with a constant stretching rate of 60 mm/min. Detailed experimental design and results appear in Supplementary Fig. 26.

Electrochemical characterization of PLCL/PP electrodes. The electrochemical impedance and cyclic voltammogram (CV) were measured with a potentiostat (Compactstat, Ivium Technologies, The Netherlands) in PBS (1X, pH 7) at 37 °C. PLCL/PP with various compositions (electrode area, ~1 mm^2), a wire-type platinum electrode, and Ag/AgCl (3 M KCl) electrode were used as working, counter, and reference electrodes, respectively. The impedance was scanned with a sin wave (frequency, 1 Hz ~ 10 kHz) and an amplitude of 10 mV, while the CV was measured at a scan rate of 20 mV/s (vs. Ag/AgCl) in a range of -0.6 V to 0.6 V. The charge storage capacity (CSC) was calculated by dividing the integral area of the CV curve below 0 V by the electrode area. For electrochemical stability test, PLCL/PP electrode (0.6:0.4:1) was soaked in PBS (pH 7) at 37 °C, and CSC values were measured weekly.

Implantation of the device. We used 20 Sprague-Dawley rats (male, 6 weeks old, 180 ~ 200 g) that were purchased from Orientbio (South Korea). The animals were stabilized for 1 week, bred for more 6 weeks to gain a weight to ~550 g for unchanged heart size, and randomly divided into 5 groups: control and 1-, 2-, 4-, and 8-weeks. For implantation of the cardiac jackets, animals were anesthetized with isoflurane (2 % in oxygen). Endotracheal intubation was conducted using a 16G intraveneous catheter, and the animals were connected to a mechanical ventilator (Model 683, Harvard Apparatus) and placed supine, followed by removal of chest hair and disinfection with povidone-iodine. Then, the heart was exteriorized via a sternotomy, and the pericardium was removed. The cardiac jacket was worn over the heart in the suture-free manner described in Supplementary Fig. 27, and the stingray barb-inspired threads were trimmed. A surgical adhesion barrier (Medicurtain, Shin Poong Pharmaceutical, South Korea) was smeared entirely on the implanted site to promote wound healing, anti-inflammatory action, and prevention of adhesion with surrounding tissues. The thoracic cavity, muscle, and skin were sequentially sutured by using 3-0 silk suture (AILEE, South Korea). Here, the pin connector was left in the subcutaneous area, exposing pins out of the skin, and a custom-made jacket was worn on the rats to protect the pins. The animals were placed on a heating pad and ventilated with 100 % oxygen until autonomous breathing was recovered. To alleviate pain and infection, antibiotics (10 mg/kg, cefazolin, Chong Kundang, South Korea) and analgesics (5 mg/kg, ketorolac tromethamine, DongKwang pharm, South Korea) were administered intramuscularly for 3 days (once a day) after the surgery.

In vivo EP/strain sensing and electrical stimulation. All experiments were performed by anesthetizing animals with isoflurane (2 % in oxygen). For measuring surface ECG, commercial gel electrodes were attached to the skin of the right foreleg, the left hindleg, and the left foreleg of a rat as anodic, cathodic, and ground electrodes, respectively. PLCL/PP

electrodes placed on the right atrium (RA) and left ventricle (LV) were used to record epicardial ECG. Surface and epicardial ECGs were collected at a sampling frequency of 1 kHz with the NI DAQ and the amplifier and filtered using Matlab, similar to the measurement processes in Fig. 3d. Myocardial strain was measured with PLCL/Mo placed on LV by recording a resistance with the source meter at a sampling frequency of 1 kHz. For evaluating the capability of pacemaking, two PLCL/PP electrodes placed on LV were used to apply an electrical field with a pulse (pulse width, 0.1 ms; pulse interval, ~142 ms) and an amplitude of 5 V by a pulse stimulator (Model 2100, A-M Systems, USA), while simultaneously monitoring epicardial ECG.

Hemodynamic studies. Hemodynamic analysis was performed immediately prior to sacrifice. Briefly, after calibrating a pressure-volume catheter (ADV550, Transonic Systems, USA) in normal saline, the probe was inserted into the left ventricle of the rat heart, and left ventricular pressure-volume relation was recorded by Power Lab 16/30 (AD Instruments, USA). Then, cardiac dimensional and functional characteristics were analyzed from at least 5 consecutive cardiac cycles within baseline normal recordings using LabChart™ software (AD Instruments, USA).

Histology analysis and blood test. Monitoring the changes in tissue morphology and inflammation at the implanted sites was conducted during 8 weeks of device implantation. After the sacrifice of animals, cardiac tissues were fixed by immersing in 4 % paraformaldehyde (PFA), embedded in a paraffin block, sectioned into a thickness of 4 μm, underwent hematoxylin and eosin staining, and photographed using a slide scanner (Axioscan 7, ZEISS, Germany). At 0, 4, and 8 weeks of implantation, approximately 3 mL of blood was collected from animals (Sprague-Dawley, n=3) into ethylenediaminetetraacetic acid (EDTA)-

and gel-tubes to evaluate blood counts and blood chemistry, respectively. NEODIN BioVet Laboratory (South Korea) conducted the assays.

Supplementary Note 11. Effect of temperature on the sensitivity of PLCL/Mo composite in physiological condition

The resistance of PLCL/Mo (9:1) composite increases as the temperature rises from 35 °C to 39 °C, primarily due to the decrease in conductivity of Mo flakes and the thermal expansion of PLCL matrix (See the Supplementary Fig. 32a). And, the PLCL/Mo composite showed a similar temperature-independent change in resistance (R – R0), at 0.05 % strain generated by cardiac movements, which was estimated by comparison of Supplementary Figs. S15 and S30 (See the Supplementary Fig. 32b). Separation of these variables might be achieved through use of low thermal conductive materials and encapsulation with thermally-insulative materials. If a temperature sensor was additionally integrated into the cardiac jacket, the temperature change caused by heart malfunctions could be monitored and used for calibrating output signals.

Supplementary Figures

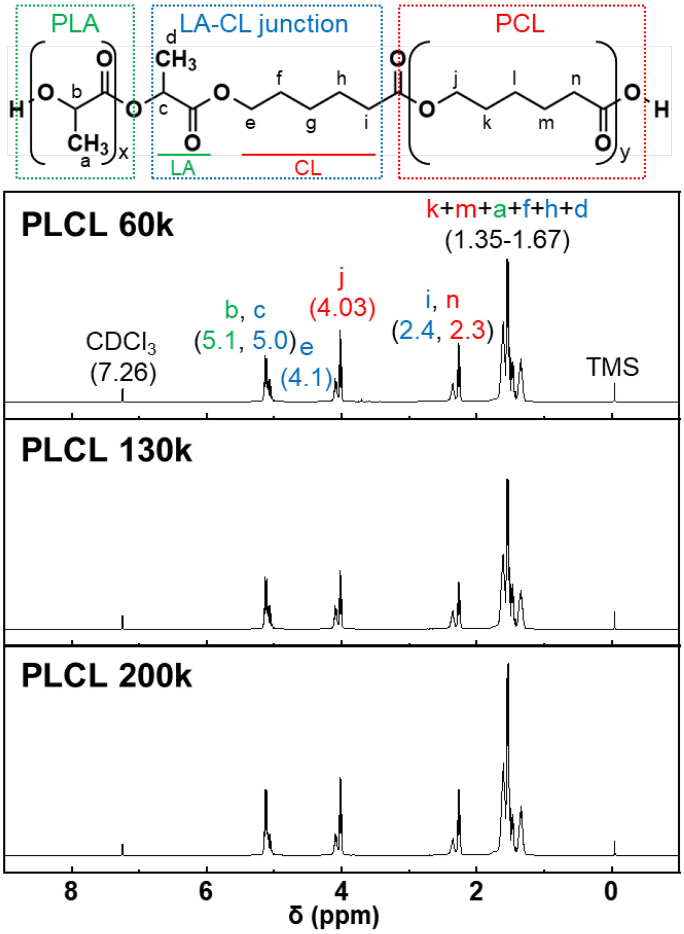

Supplementary Figure 1. ^{1}H nuclear magnetic resonance (NMR) spectra of synthesized PLCL elastomers with molecular weights of 60k, 140k, and 200k.

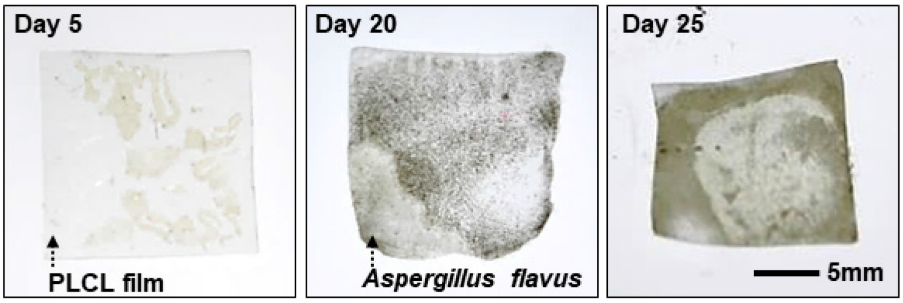

Supplementary Figure 2. Optical images of enzymatic degradation of PLCL films (140k, 100 μm-thick) by the fungus, *Aspergillus flavus*, at 37 °C (relative humidity (RH): 50 %). Similar results were obtained in three independent experiments.

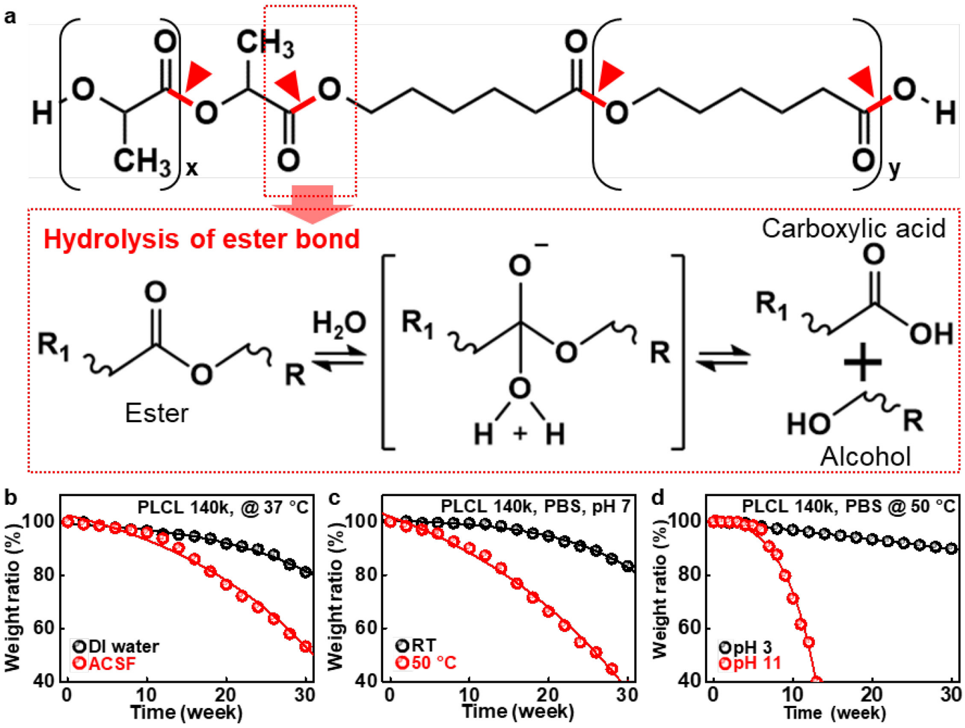

Supplementary Figure 3. (a) Hydrolytic degradation mechanism of PLCL, where chain cleavage of ester bonds via hydrolysis generates biocompatible lactic and caproic acids (carboxylic acid) as final products[1,2]. (b-d) Time-dependent changes in weight ratio of PLCL film (140k, 100 μm-thick) as a function of type (b), temperature (c), and pH levels (d) of solution. Variation in an ionic content/concentration affected the hydrolytic reaction, which caused rapid dissolution rates in artificial cerebrospinal fluid (ACSF) than deionized water, while high temperature and pH values accelerated the dissolution of PLCL.

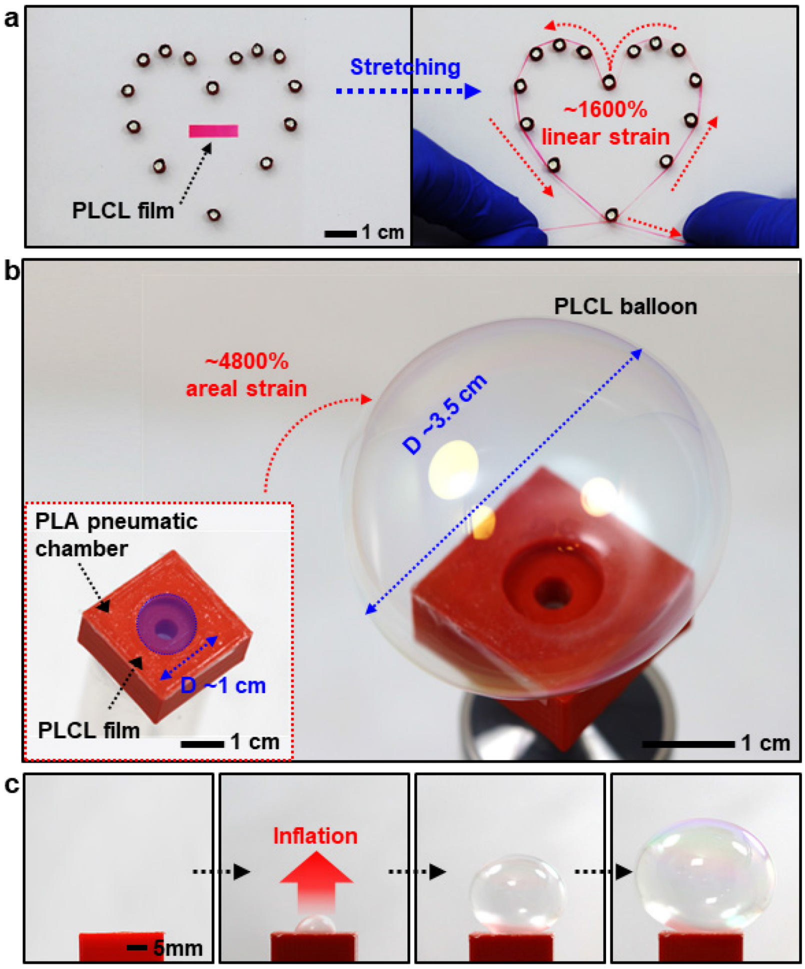

Supplementary Figure 4. Balloon inflation of PLCL. (a) Demonstration of an ultra-stretchable performance of PLCL film (60k, 100 μm-thick) before (left) and after (right) an extreme elongation (similar to ~1600 % linear stretching) using a heart-shape structure. (b) Balloon-like inflation of a PLCL membrane (140k, 100 μm-thick) equivalent to ~4800 % areal strain, and an image of the membrane attached to 3D-printed PLA chamber with a hole connected to pneumatic source before the inflation. (c) Sequential images of inflation of the PLCL membrane.

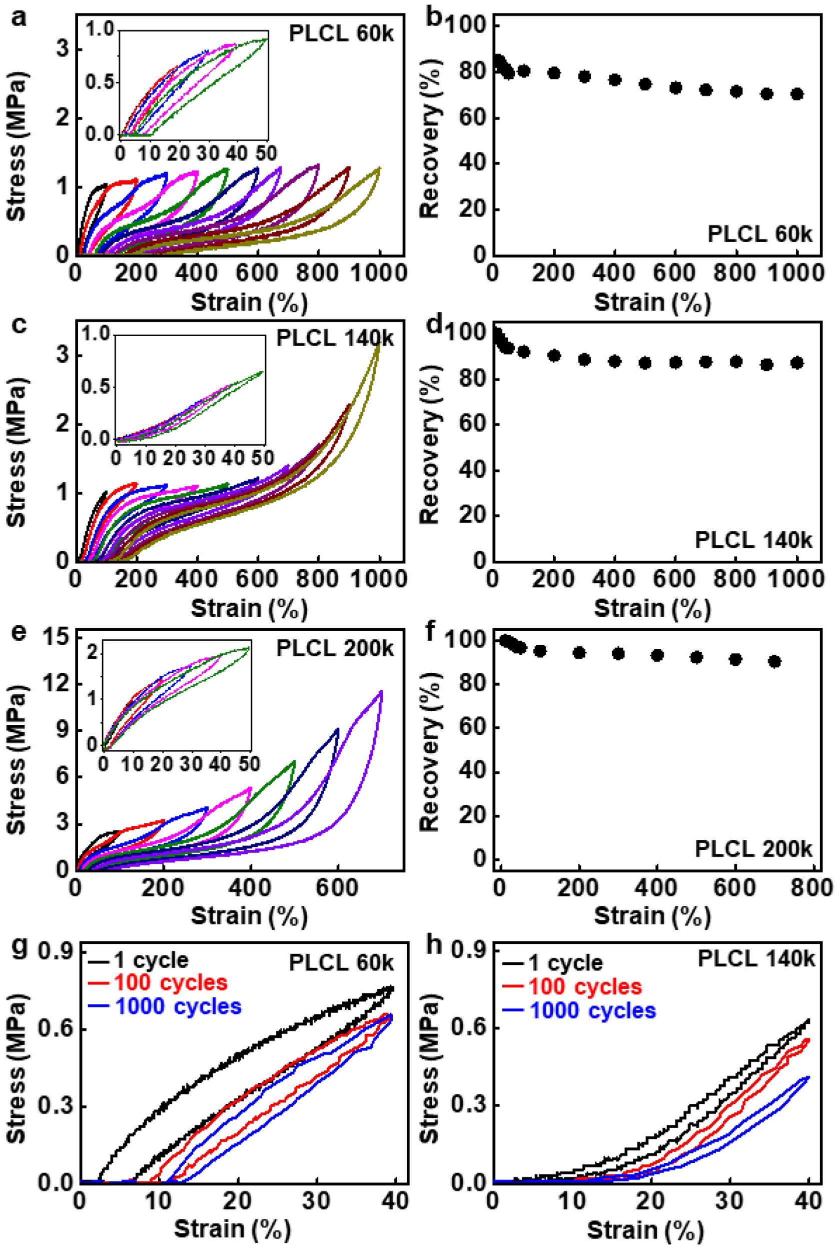

Supplementary Figure 5. Mechanical properties of PLCL. (a-f) Strain cycling behaviors and strain recovery rates of PLCL elastomers with different molecular weights of 60k (a,b), 140k (c,d), and 200k (e,f). (g,h) Cyclic durabilities of 60k (g) and 140k (h) at a strain of 40 %.

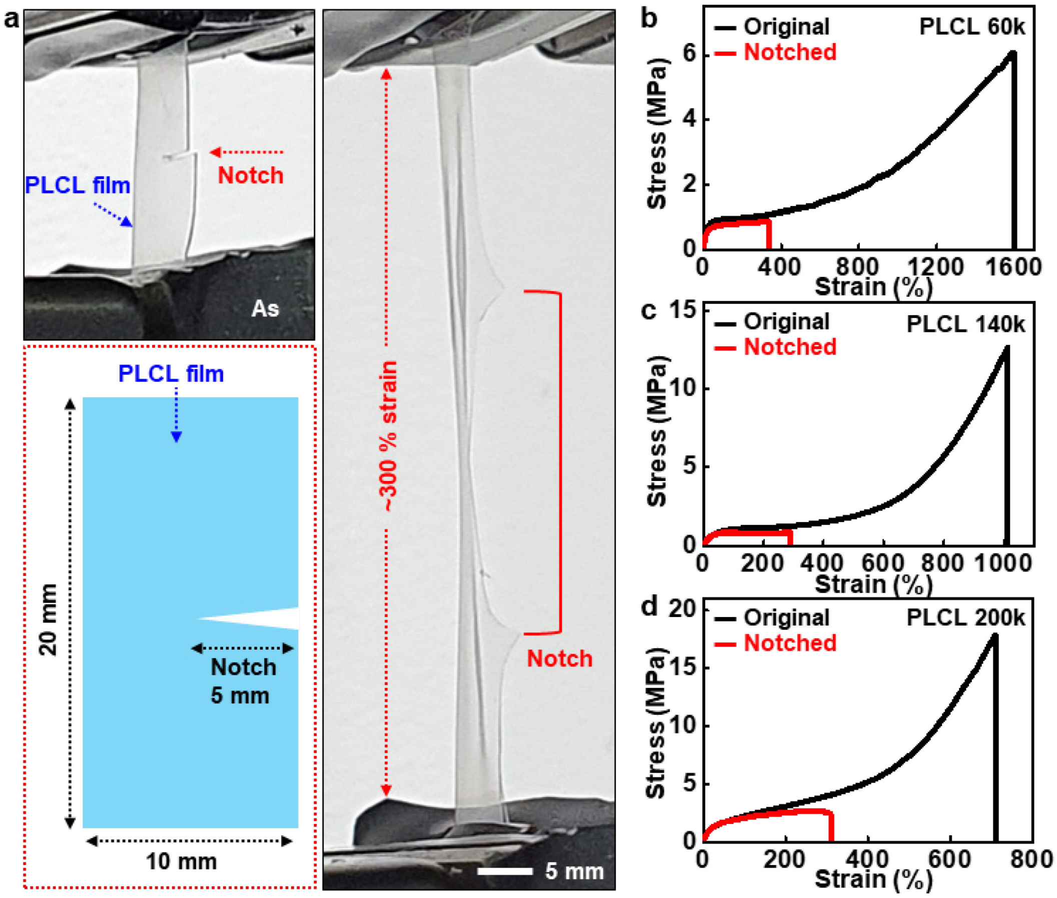

Supplementary Figure 6. High toughness and fracture tolerance of PLCL. (a) Schematic illustration of a PLCL film with notch and images of a notched PLCL film (200k, 100 μm-thick) before and after 300 % stretching. (b-d) Stress-strain curves of the original and notched PLCL films with different molecular weight of 60k (b), 140k (c), and 200k (d).

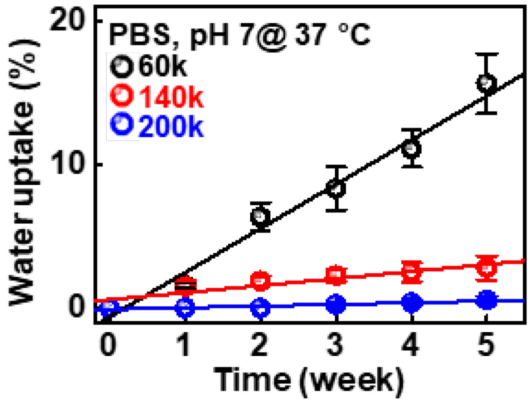

Supplementary Figure 7. Time-dependent swelling ratio of PLCL films (60k, 140k, and 200k, 100 μm-thick) in PBS (pH 7) at 37 °C. Data are presented as mean values +/- standard deviation. n=3 independent samples per group.

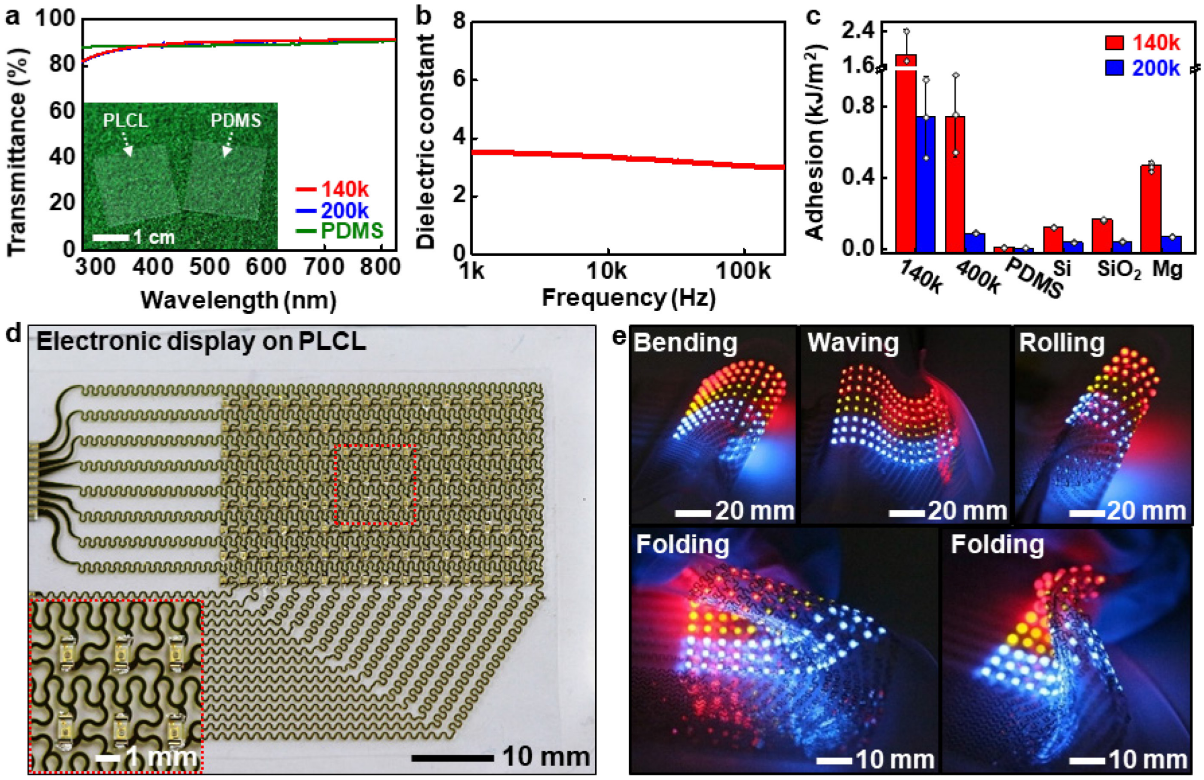

Supplementary Figure 8. (a) Optical transmittance of thin films (100 μm-thick) of PLCLs (140k and 200k) and polydimethylsiloxane (PDMS), with their images in the inset. (b) Dielectric constant of PLCL as a function of frequency. (c) Adhesion energy between bonded pieces of PLCL (140k and 200k) and between PLCL (140k and 200k) and other materials, including PDMS, Si, silicon dioxide (SiO_2), and magnesium (Mg). Data are presented as mean values +/- standard deviation. n=3 independent samples per group. (d) Flexible electronic display (8x16 LED matrix array) on PLCL substrate and the magnified image of LEDs (inset). (e) Images of the electronic display upon various deformation modes, such as bending, waving, rolling, and folding.

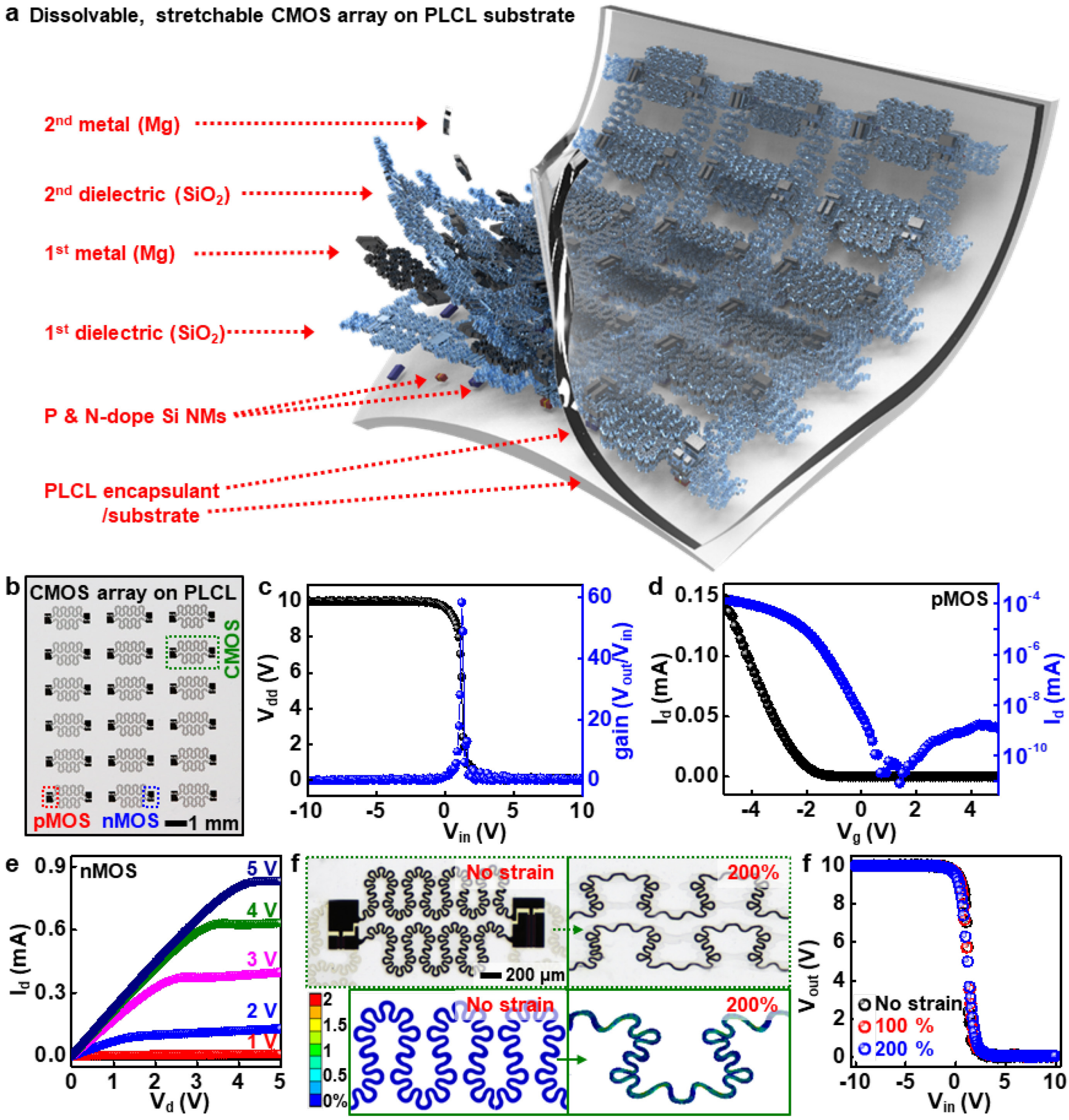

Supplementary Figure 9. Fully-dissolvable, stretchable complementary metal-oxide-semiconductor (CMOS) inverter array on PLCL. (a,b) Schematic exploded view (a) and optical image (b) of the CMOS array on PLCL substrate. (c) Output voltage characteristics of a representative CMOS inverter at V_{dd} = 10 V. The gain was ~60. (d) Linear (black) and log scale (blue) transfer curves of a typical p-channel metal-oxide-semiconductor field-effect transistor (MOSFET). The channel length and width were 5 μm and 300 μm, respectively. The mobility (linear regime) and on/off ratio were ~78 $cm^2/V \cdot s$ and ~106, respectively. (e) I-

V characteristics of a typical n-channel MOSFET. The channel length and width were 20 μm and 100 μm, respectively. The mobility (linear regime) and on/off ratio were ~310 $cm^2/V\cdot s$ and ~105, respectively. (e) Optical images and the corresponding mechanical simulations of the CMOS with (left) and without (right) a uniaxial strain of 200 %. (f) Output voltage characteristics of a CMOS inverter (V_{dd} = 10 V) with tensile strains of 0, 100, and 200 %.

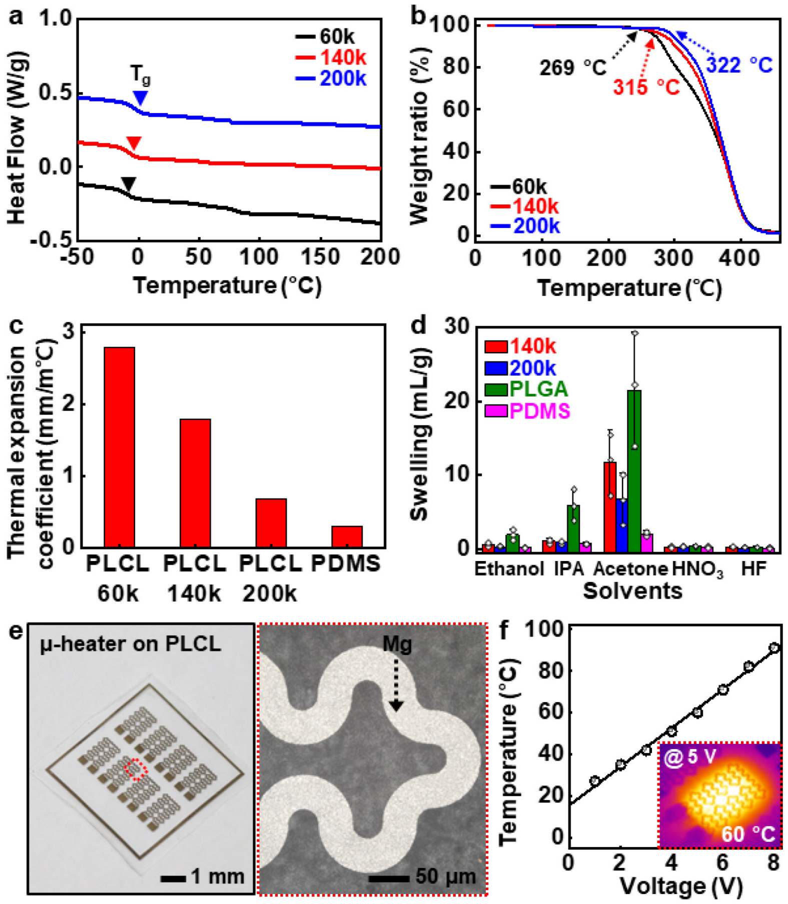

Supplementary Figure 10. (a) Differential calorimetry scanning (DSC) plots of PLCLs (black, 60k; red, 140k; blue, 200k). (b) Thermogravimetric analysis (TGA) thermograms of PLCLs (black, 60k; red, 140k; blue, 200k). (c) Thermal expansion coefficient of PLCLs (60, 140, and 200k) and PDMS. (d) Solvent compatibility of PLCLs (red, 140; blue, 200k), polylactide-co-glycolide (green, PLGA) as a widely-used biodegradable polymer, and PDMS (magenta), to various organic solvents, such as ethanol, isopropanol (IPA), acetone, nitric acid (HNO3), and hydrofluoric acid (HF) at room temperature. Data are presented as mean values +/- standard deviation. n=3 independent samples per group. (e) An array of Mg microheaters directly fabricated on PLCL substrate (left) by photolithography and lift-off processes, and its magnified view image (right). The metal pattern exhibited sharp corners and edges of small features without cracks. (f) Voltage-dependent heating performance of the microheater (9 °C/V), with a thermogram of the microheater operated at 5 V in the inset.

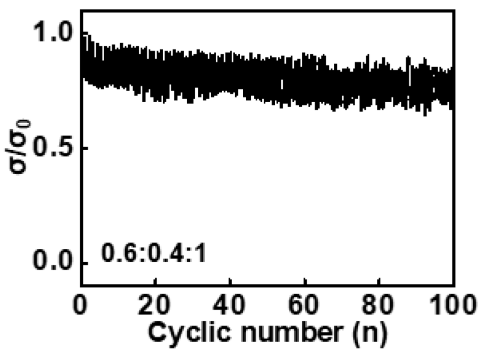

Supplementary Figure 11. Cyclic strain stability of PLCL/PP elastic conductor (0.6:0.4:1) for 40 % stretching.

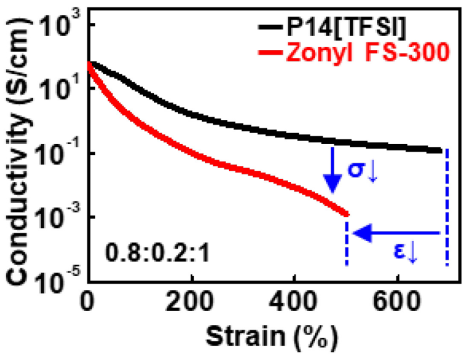

Supplementary Figure 12. Strain-dependent conductivity changes of PLCL/PP (0.8:0.2:1) with different additives of P14[TFSI] and Zonyl FS-300.

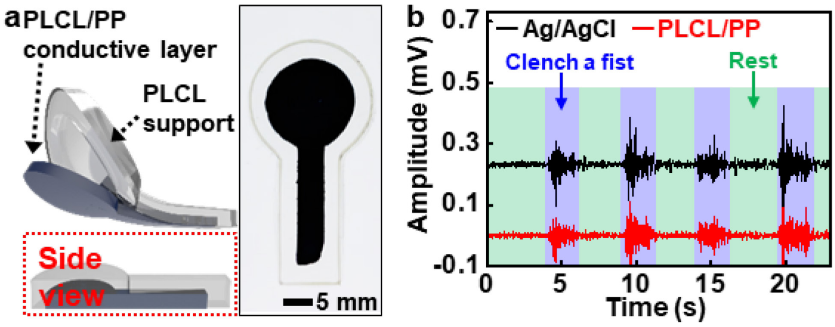

Supplementary Figure 13. (a) Schematic illustration (left) and optical image (right) of a PLCL/PP probe for measuring electrophysiological (EP) signals. (b) Measurements of electromyogram (EMG) using the probes and commercial Ag/AgCl electrodes.

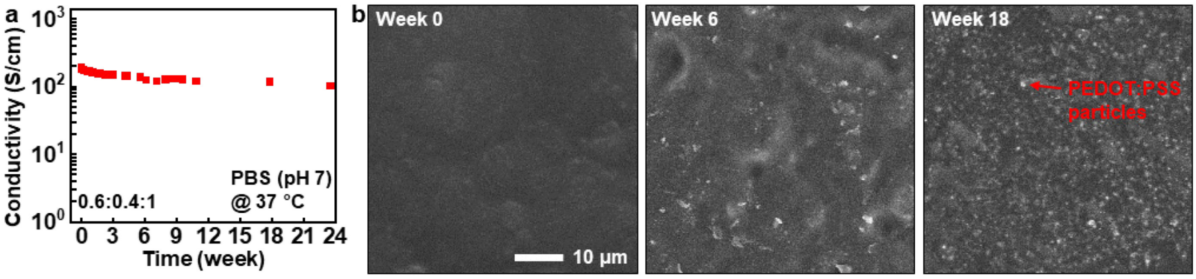

Supplementary Figure 14. Dissolution behaviors of PLCL/PP. Changes in conductivity (a) and microstructure (b) of PLCP/PP (0.6:0.4:1) as a function of immersion time in PBS (pH 7) at 37 °C.

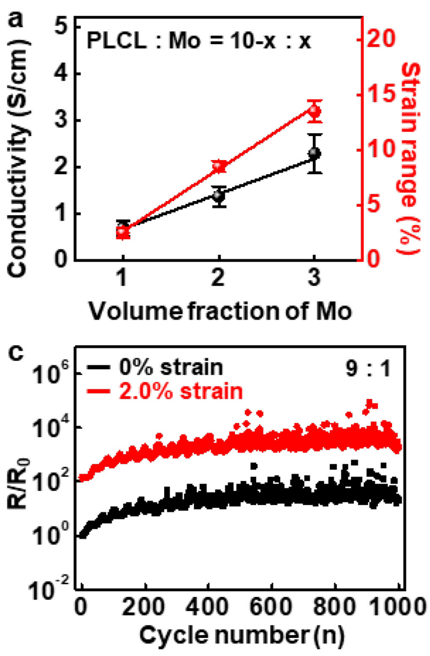

Supplementary Figure 15. Electrical characteristics of PLCL/Mo. (a) Various conductivities and strain ranges of PLCL/Mo with different volume fractions of Mo. Data are presented as mean values +/- standard deviation. n=3 independent samples per group. (b) Reversible loading-unloading behavior of PLCL/Mo (9:1) for 2.0 % strain. (c) Cyclic durability test of PLCL/Mo (9:1) with 2.0 % strain. (d) Cyclic loading-unloading behaviors of the PLCL/Mo (9:1) for various tensile strains of 0.01, 0.03, 0.05, and 0.1 %, and the magnified plot for 0.01 % strain in the inset.

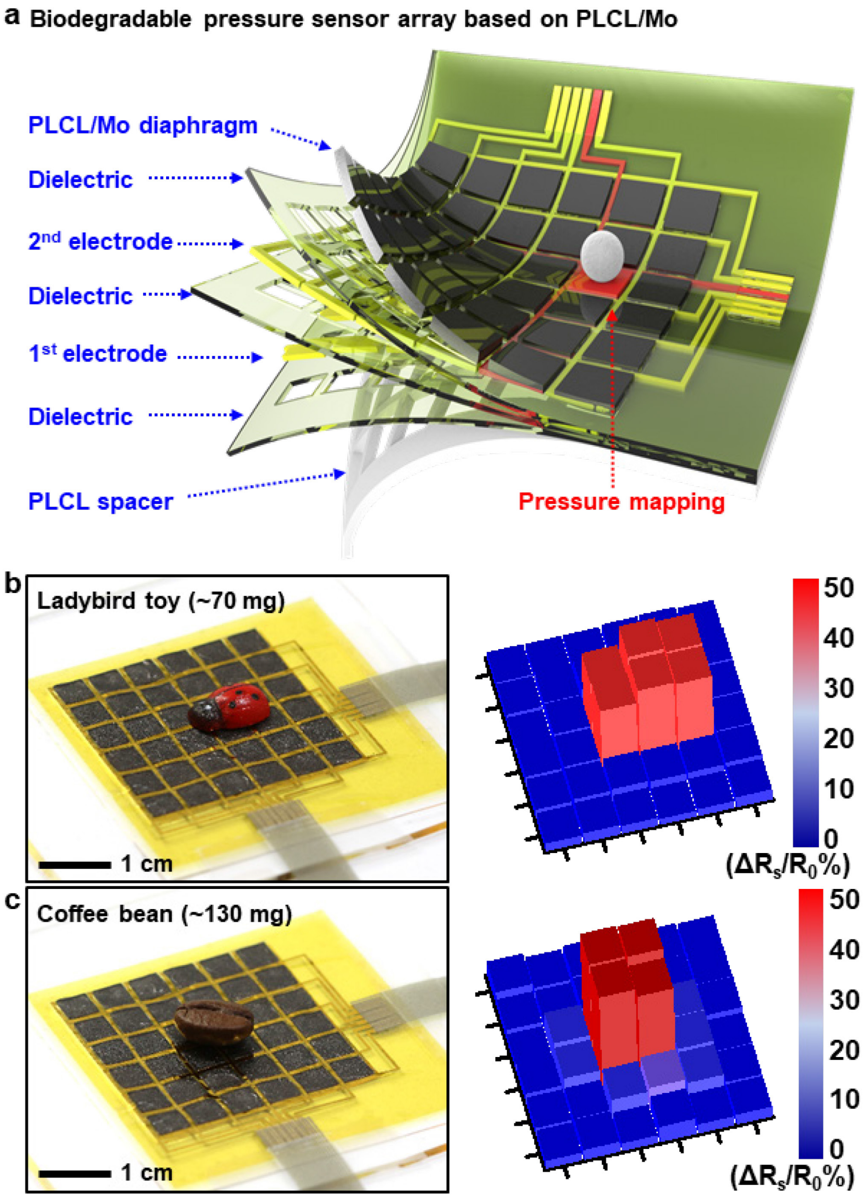

Supplementary Figure 16. Biodegradable multipixel pressure sensor array based on PLCL/Mo. (a) Schematic illustration of PLCL/Mo-based biodegradable pressure sensor array. (b,c) Measurements of pressure distributions of a ladybird toy (~70 mg) (b) and a coffee bean (~130 mg) (c) by the sensor array.

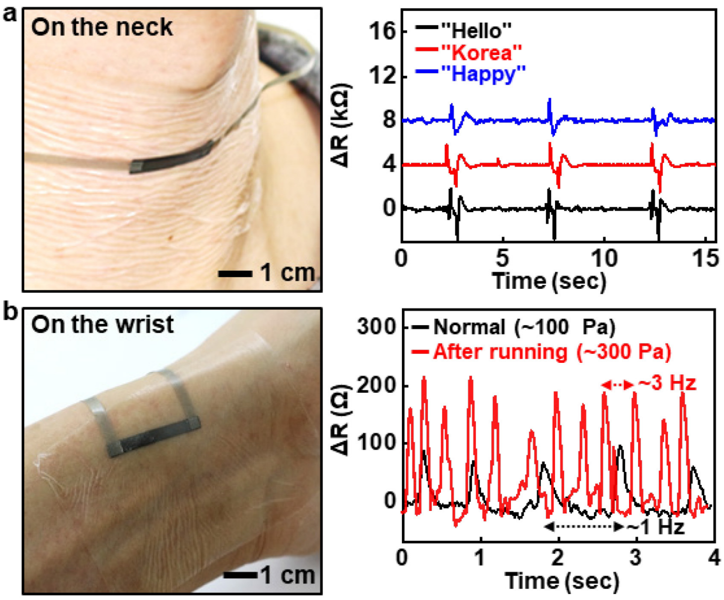

Supplementary Figure 17. Applications of highly strain-sensitive PLCL/Mo. (a) Image of a PLCL/Mo (9:1)-based strain sensor attached to the neck, and human speech recordings for three different words. (b) Detection of heart rates before and after running by the sensor placed on the wrist.

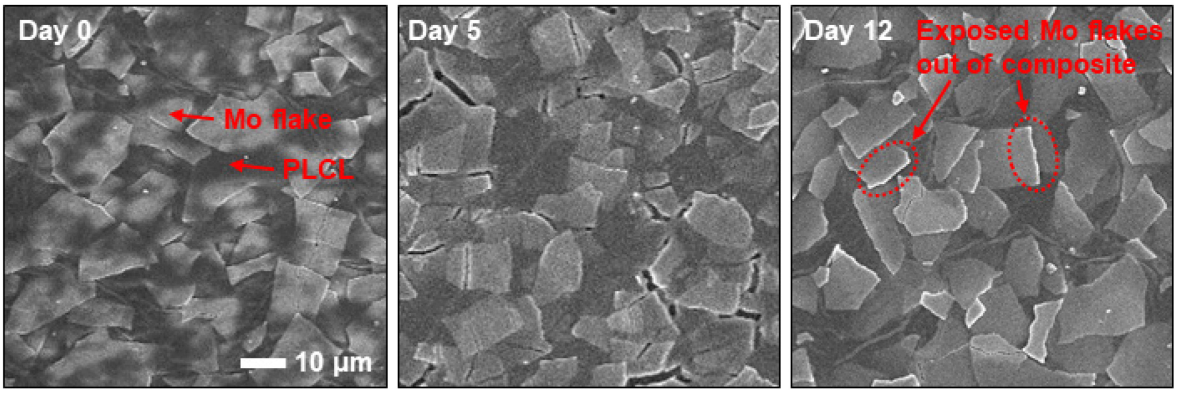

Supplementary Figure 18. Dissolution behaviors of PLCL/Mo (9:1) as a function of immersion time in PBS (pH 7) at 37 °C.

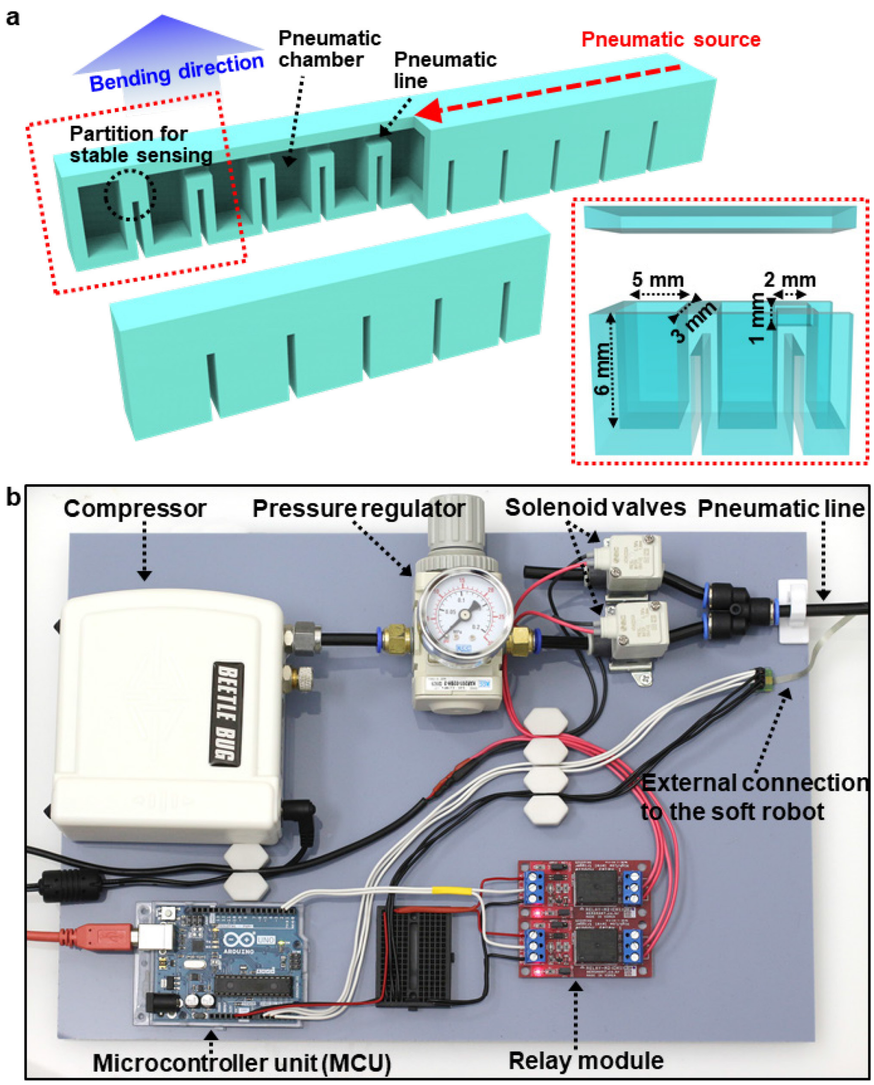

Supplementary Figure 19. (a) Schematic illustration of a PLCL-based gripper consisting of 10 pneumatic small chambers with an isolated chamber for stable sensing. When a pneumatic source was applied, the chambers were inflated to cause the gripper to bend. We isolated a pneumatic chamber of actuators, on which the sensors are embedded, from an applied pneumatic pressure, and employed Mg serpentine traces and strain-tolerant PLCL/PP for electrodes to retain electrical connections during actuation. (b) Photograph of custom-made pneumatic pressure-generating system integrated with a microcontroller. Pressure regulator (KAR201 series, KCC, Korea); Solenoid valve (VDW22GA, SMC, Korea); Relay module (NER-13705, Nero Tech, Korea); Microcontroller unit (Arduino UNO, Arduino, Italy); Compressor (Beetle bug, Denkou Syounendan, Japan).

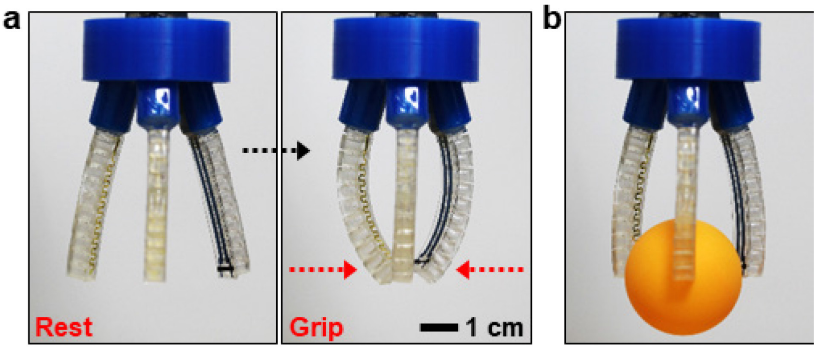

Supplementary Figure 20. Actuation of the smart soft gripper. (a) Actuation behavior of the gripper. (b) The gripper holding a ping pong ball.

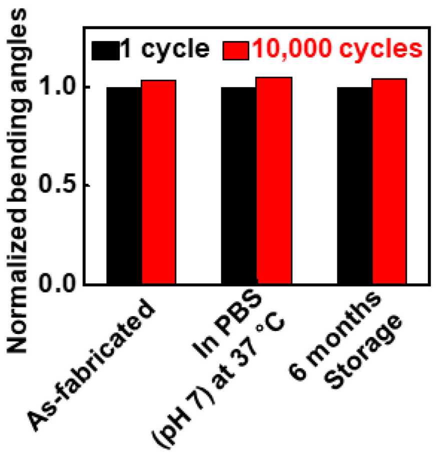

Supplementary Figure 21. Cyclic operational stability of the gripper with a pneumatic pressure of 55 kPa under various conditions.

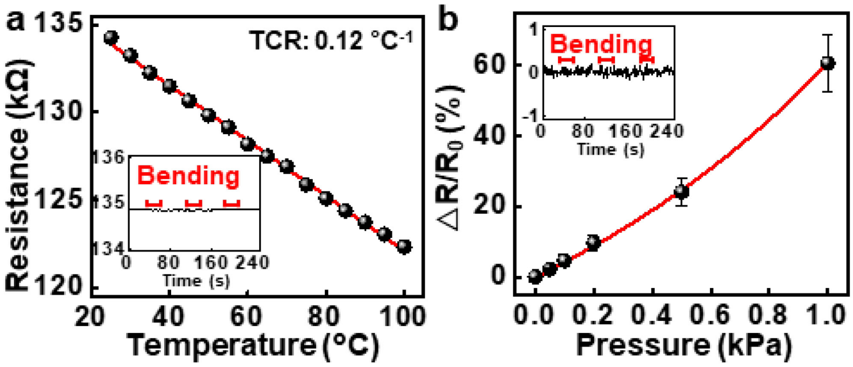

Supplementary Figure 22. Detection sensitivity and sensing stability (during repeated actuation with a bending angle of 90 °) of the temperature (a) and pressure (b) sensors. Data are presented as mean values +/- standard deviation. n=3 independent samples.

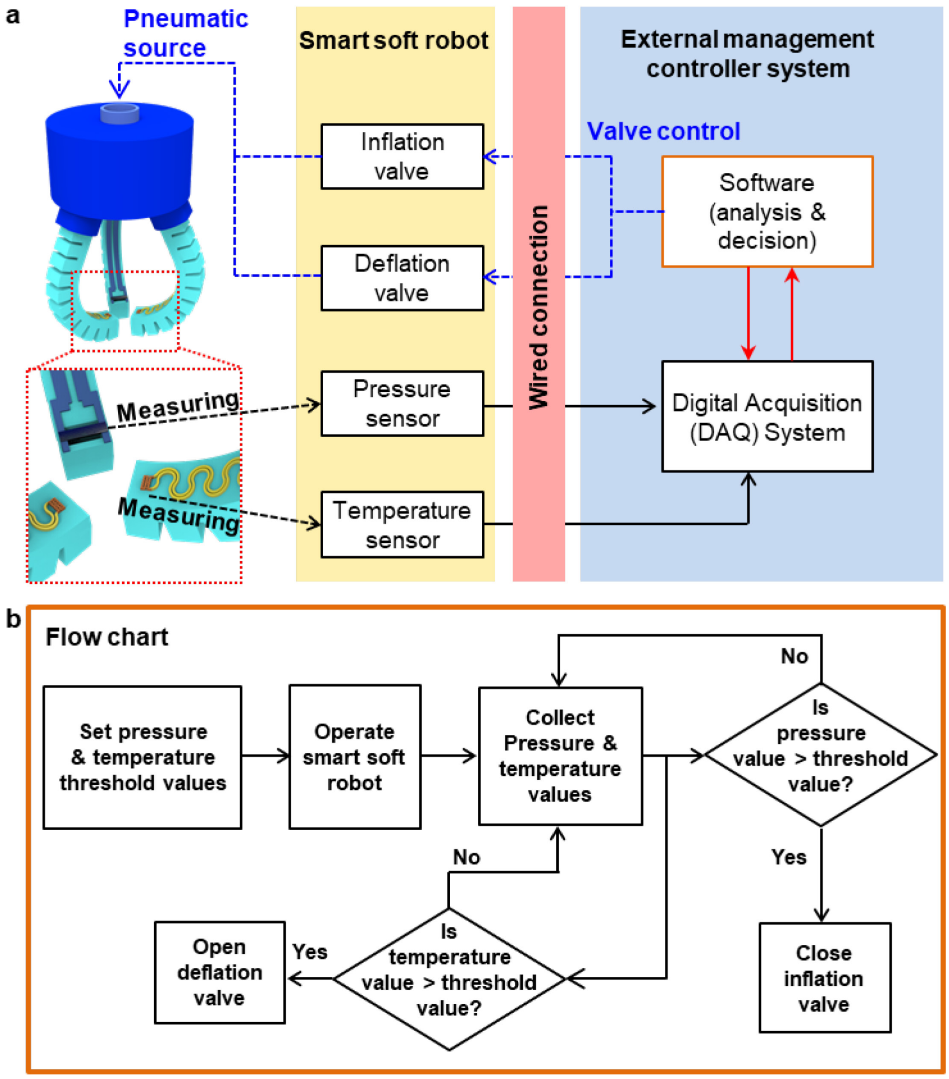

Supplementary Figure 23. Data acquisition procedure and customized algorithm for management of gripper function. (a) Operational block diagram of the overall system for data acquisition and manipulation of pneumatic source. (b) Customized algorithm flowchart for perceiving temperature and pressure thresholds.

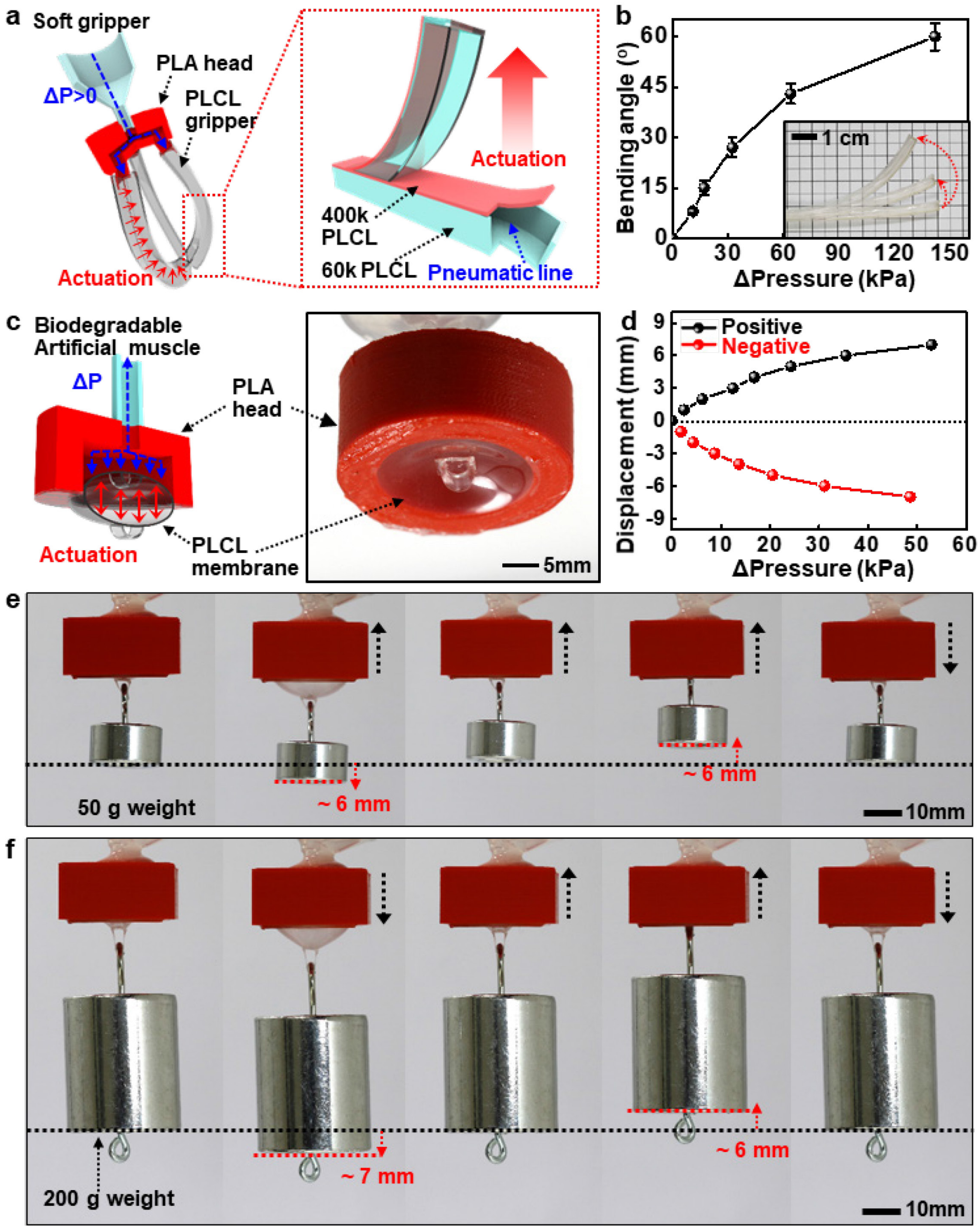

Supplementary Figure 24. Biodegradable soft grippers. (a) Schematic illustration of a biodegradable soft gripper constructed with 60k and 200k PLCL elastomers with different expansion rates. When a pneumatic source was applied, the 60k PLCL part of the gripper was inflated whereas the 200k PLCL part remained uninflated, causing the gripper to bend. (b) Correlation of the bending angle and applied pressure with the merged image at several stages of actuation of the gripper in the inset. Data are presented as mean values +/- standard deviation. n=3 independent samples. (c) Schematic illustration and optical image of a biodegradable artificial muscle based on PLCL diaphragm (200k; diameter, 20 mm; thickness,

500 μm). When negative and positive pneumatic pressures were applied, the diaphragm was actuated upwards and downwards, respectively. (d) Correlation of the displacement and applied pressure. (e,f) Images of the artificial muscle lifting up/down 50 g (e) and 200 g (f) of weights which corresponds to a payload-to-weight ratio of over 1,000.

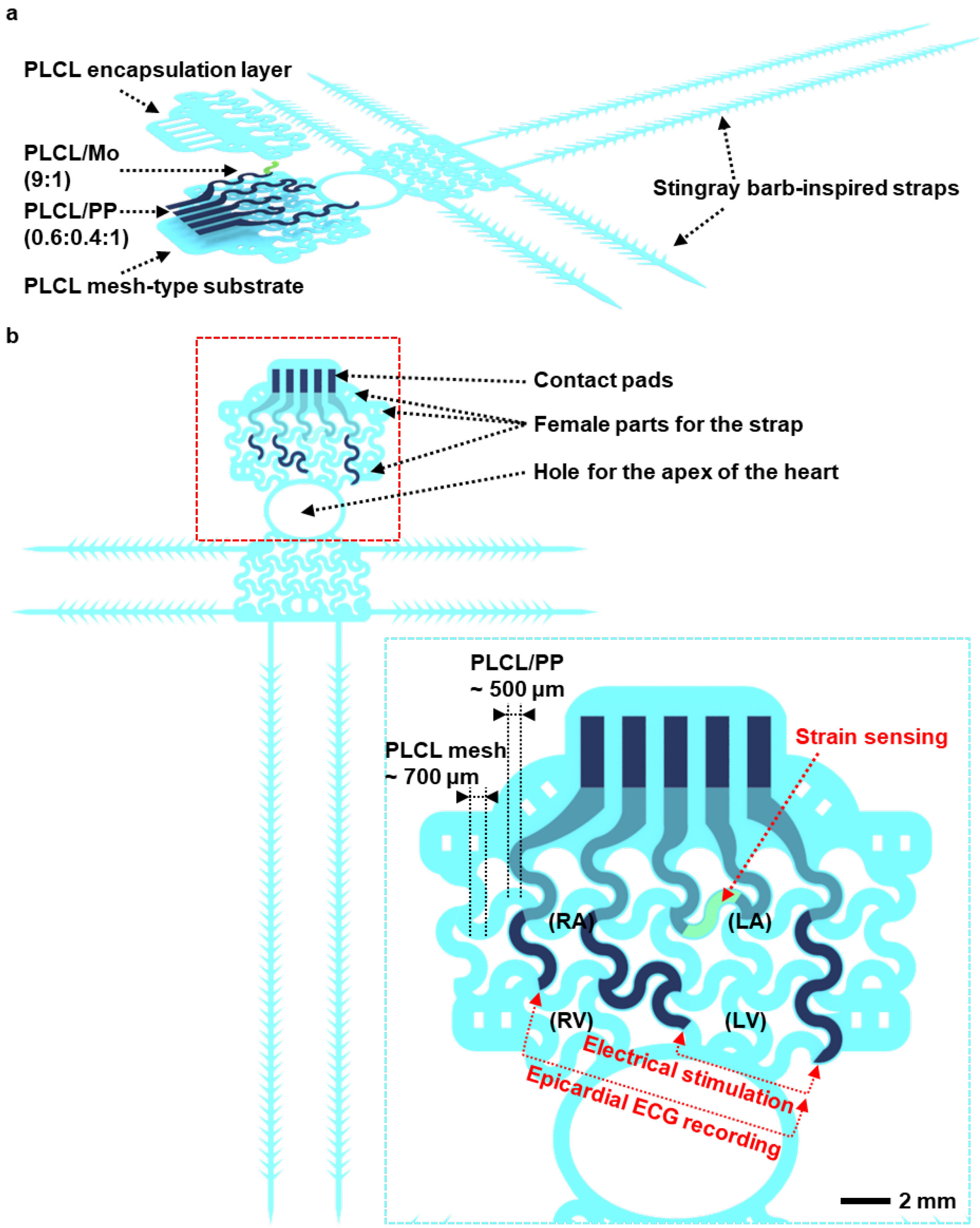

Supplementary Figure 25. Schematic exploded (a) and top (b) views of a cardiac jacket.

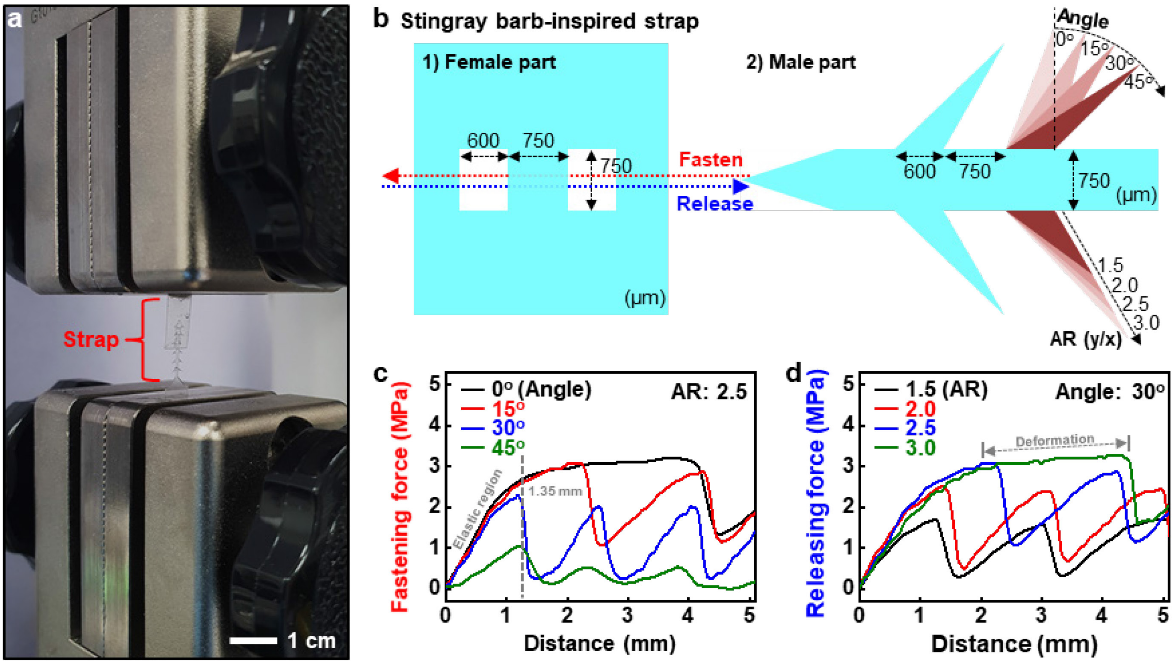

Supplementary Figure 26. A stingray barb-inspired strap for suture-free integration of the cardiac jacket to the heart. (a) Photographs of a stingray barb-inspired strap on a tensile tester. (c) Schematic description of the strap consisting of a female part and a male part with barbs of various angles and aspect ratios (AR), that can reversibly and finely tighten and loosen the cardiac jacket in increments of 1.35 mm. (d) Fastening forces of the straps with barbs of AR 2.5 and various angles, measured with a constant loading rate of 60 mm/min. The angle of 30 ° could avoid unwanted elongation of the strap, thus allowing for facile and stable fastening. The angles of 0 ° and 15 ° caused unwanted elongation, while the 45 ° angle allowed for the easy of fastening, but resulted in too low maximum releasing force. (e) Releasing forces of the straps with an angle of 30 ° and various ARs, measured with a constant loading rate of 60 mm/min. The AR 2.5 yielded the highest releasing force without significant deformation as shown in the case with AR 3.0.

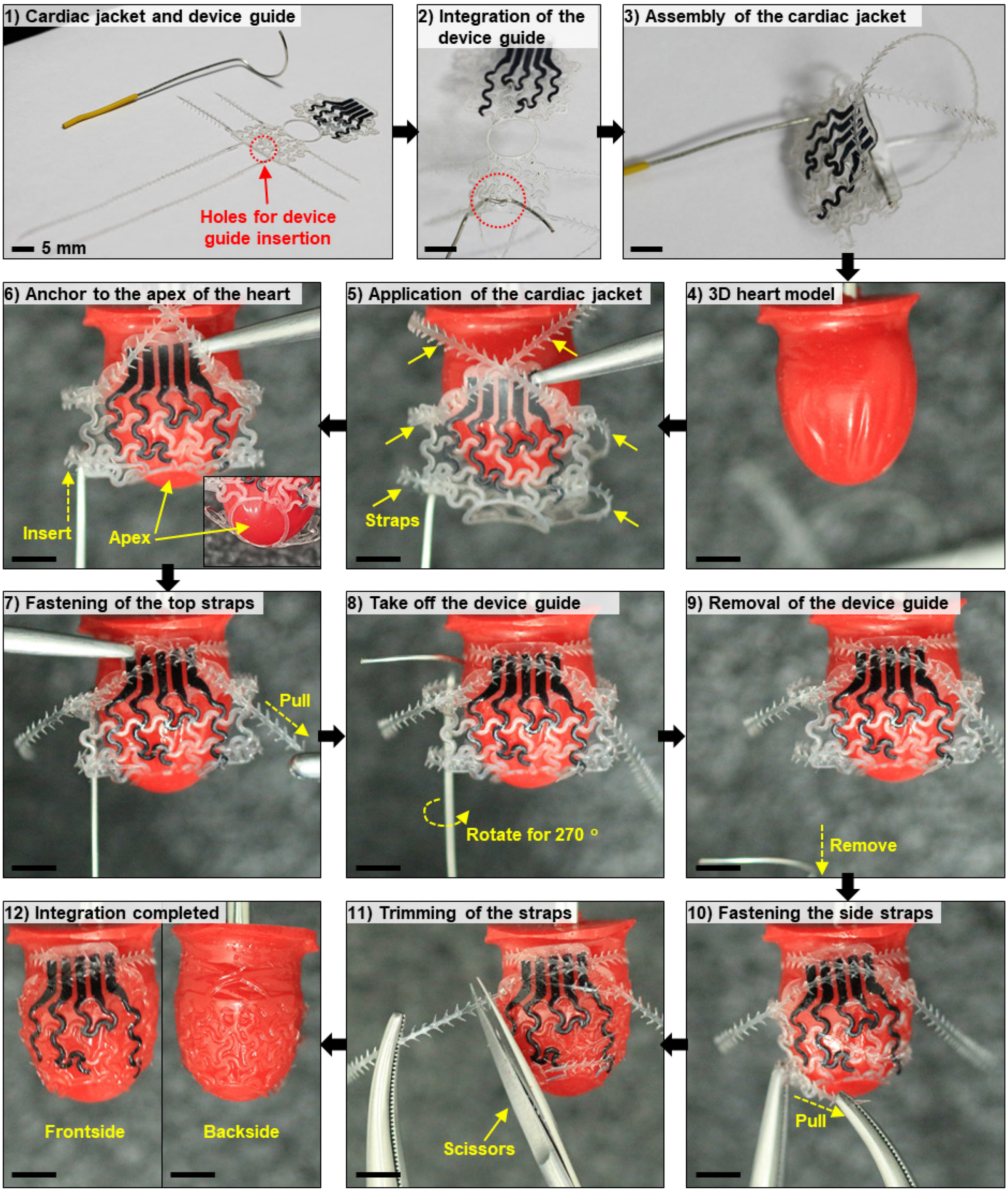

Supplementary Figure 27. Photographs of the process of putting the cardiac jacket on the 3D model of a rat heart. Thanks to the stingray barb-inspired straps and a custom-made device guide, it takes less than 5 min to complete the integration, alleviating infections/failures of a rat heart.

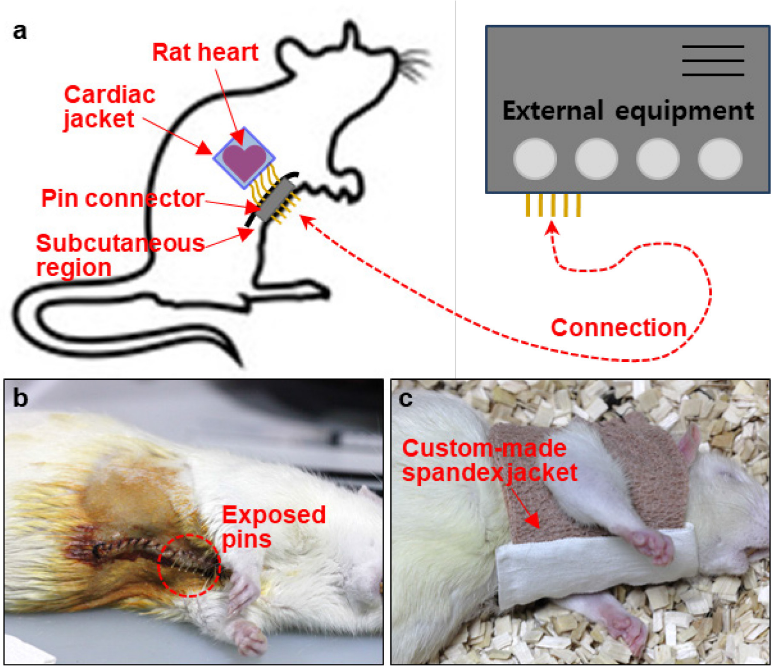

Supplementary Figure 28. (a) Schematic illustration of device connection strategy. (b) Pins exposed outside the rat chest for connection with external equipment, where the pin connector wired to the cardiac jacket was left in the subcutaneous region of the rat. (c) A custom-made spandex jacket for protecting the pins, which allowed for stable measurement/stimulation during the implantation period of 8 weeks.

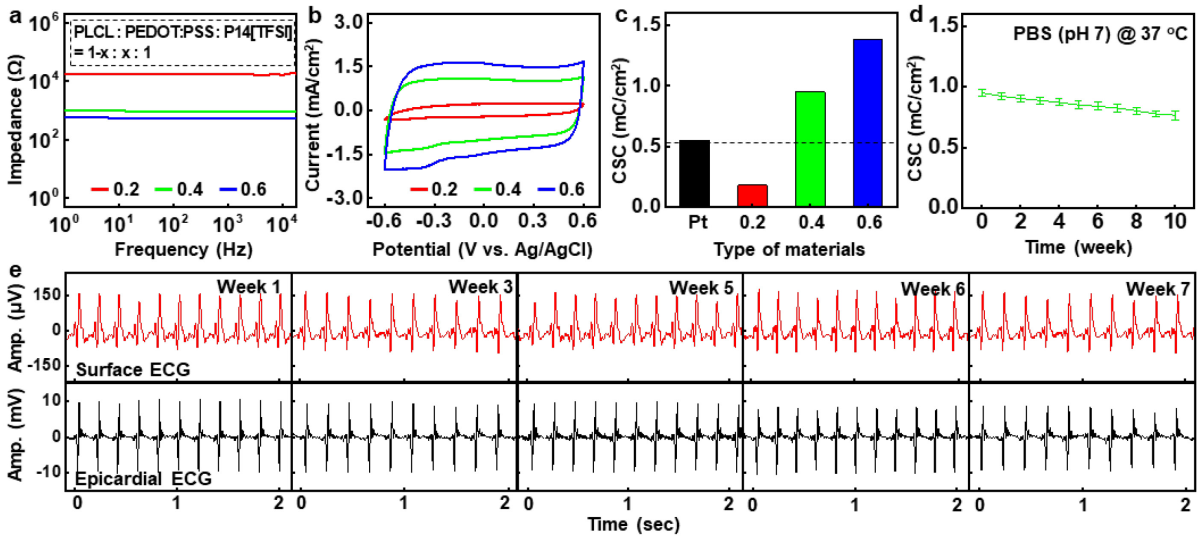

Supplementary Figure 29. (a,b) Impedance spectra (a) and cyclic voltammetry (b) of PLCL/PP with various PEDOT:PSS concentrations. (c) Charge storage capacity (CSC) of the PLCL/PPs calculated in (b) and those of platinum (Pt)[3] for comparison. (d) Long-term stability of CSC for the PLCL/PP (0.6:0.4:1) while soaking in PBS (pH 7) at 37 °C. Data are presented as mean values +/- standard deviation. n=3 independent samples. (e) Representative surface ECG signals recorded by conventional gel electrodes (top) and the epicardial ECG signals recorded by the cardiac jacket (bottom) on weeks 1, 3, 5, 6, and 7 of in vivo implantation.

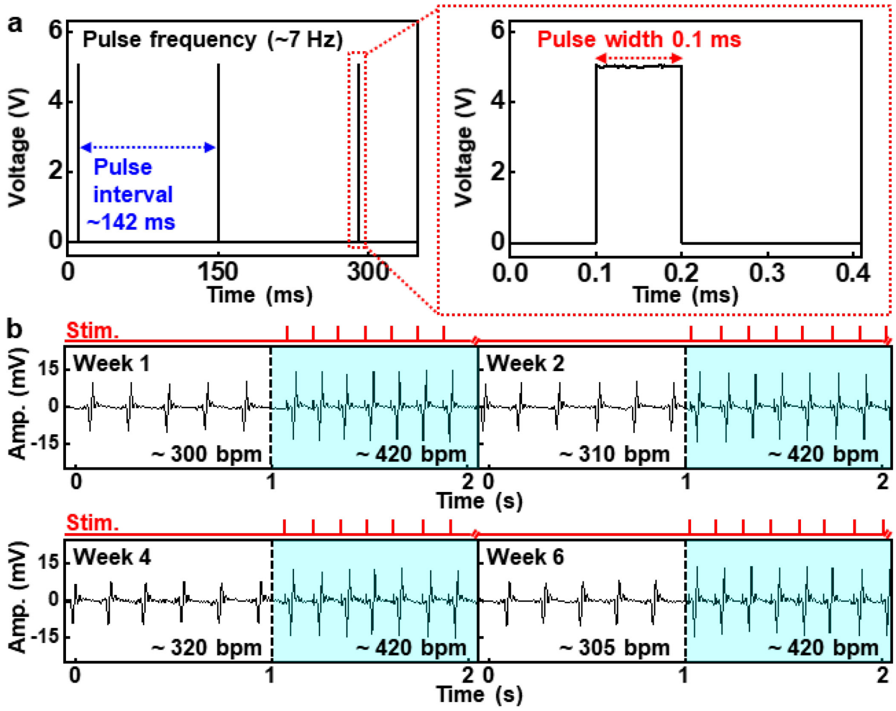

Supplementary Figure 30. (a) Time dependence of the output voltage generated by the cardiac jacket (5 V, 0.1 ms). (b) Epicardial ECG signals measured before (left white background) and during (right blue background) electrical stimulation (square wave with a duration of 0.5 ms and an amplitude of 5 V) with the implanted cardiac jacket on weeks 1, 2, 4, and 6 of implantation.

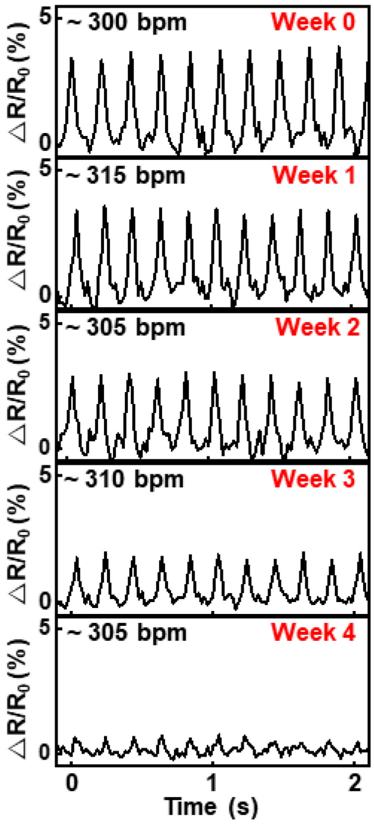

Supplementary Figure 31. Monitoring of myocardial strain with the PLCL/Mo sensor on the implanted device for 4 weeks to predict cardiac mechanics in the early stages of heart diseases. Despite the high circumferential strain of the heart (~30 %[4]), the strain applied to the sensor was estimated as low as ~0.05 %, due to the serpentine layout and high modulus (~4 MPa). A myocardial infarction, that occurred when blood flow decreases or stops to the coronary artery of the heart, results in damage of the myocardium, particularly in the left ventricle. This damage accompanies fibrosis generation, which increases the stiffness of cardiomyocytes and thus reduces the elasticity during systole/diastole. Therefore, by monitoring amplitude change of output signals detected by strain sensor, we can predict the relative stiffness of post-MI heart (vs. normal heart). Meanwhile, using a high molecular weight PLCL as encapsulation layers can extend the period of stable operation.

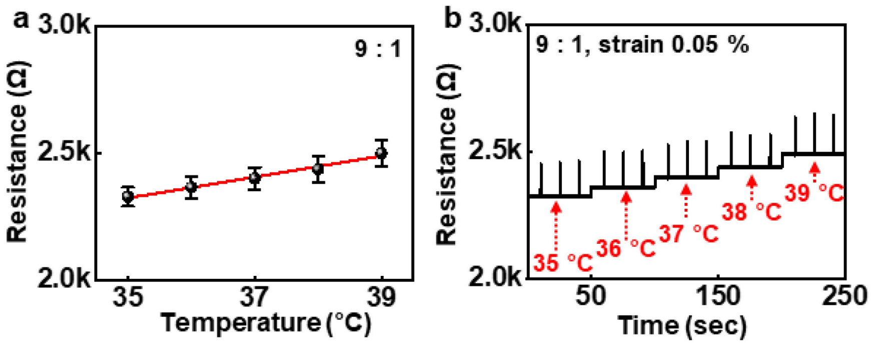

Supplementary Figure 32. (a) Base resistance changes of PLCL/Mo (9:1) as a function of temperature. Data are presented as mean values +/- standard deviation. n=3 independent samples. (b) Resistance changes of PLCL/Mo (9:1) at a strain of 0.05 % in a temperature range of 35 to 39 °C.

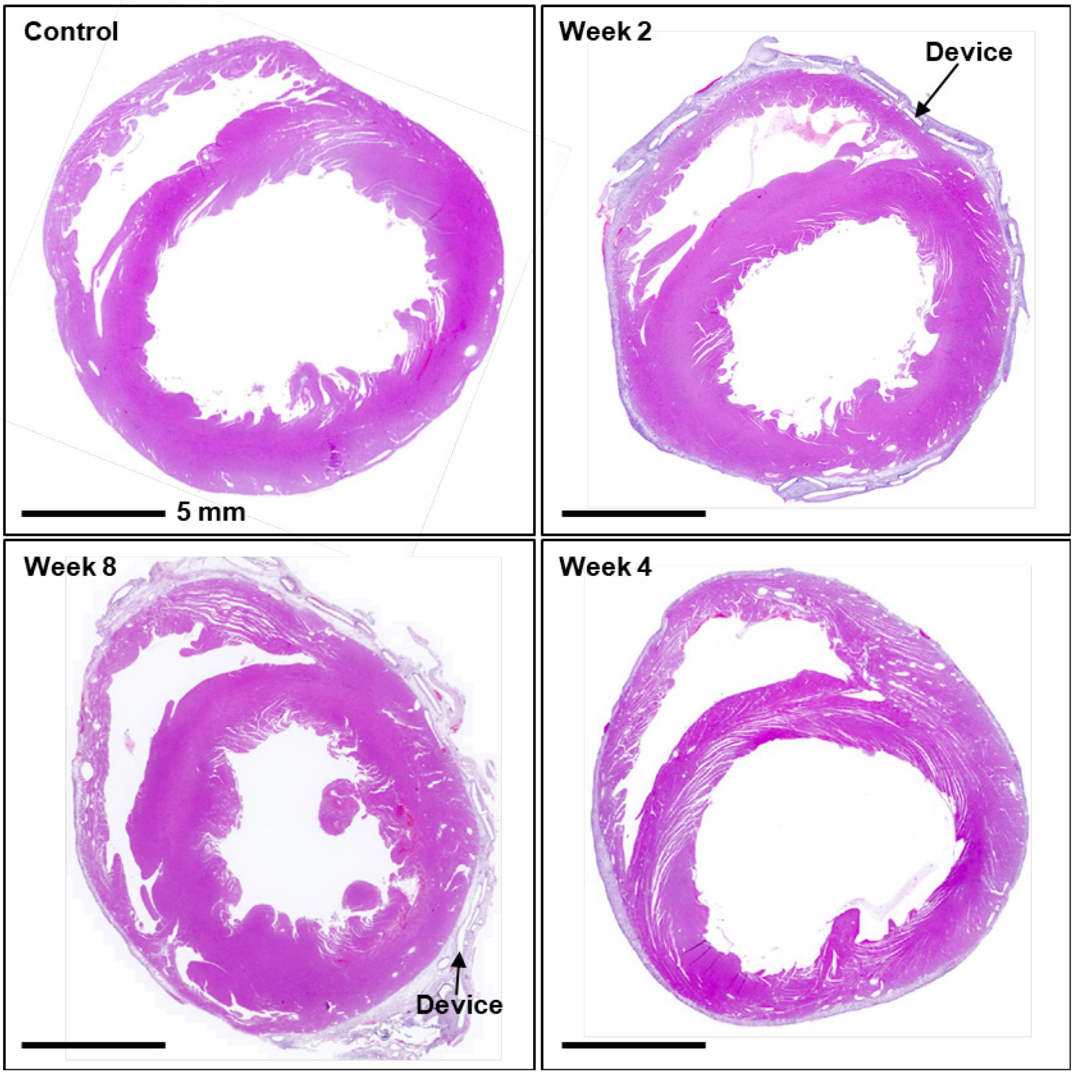

Supplementary Figure 33. Hematoxylin and eosin (H&E) staining of heart sections 0, 2, 4, and 8 weeks after device implantation.

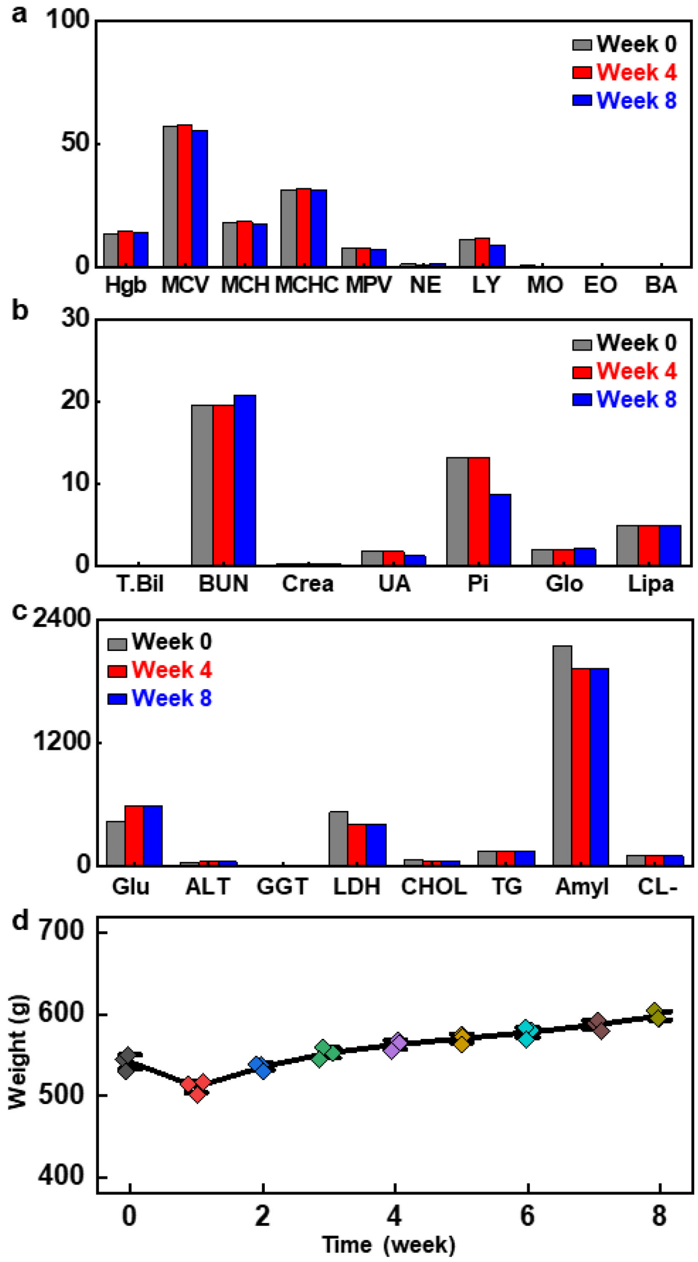

Supplementary Figure 34. (a-c) Analysis of complete blood counts (a) and blood chemistry (b,c) for rats with and without device implantation, indicating preservation of overall healthy physiology in the animals. Hgb, hemoglobin, g/dL; MCV, mean corpuscular volume, fl; MCH, mean corpuscular hemoglobin, pg; MCHC, mean corpuscular hemoglobin concentration, g/dL; MPV, mean platelet volume, fl; NE, neutrophils, K/uL; LY, lymphocytes, K/uL; MO, monocytes, K/uL; EO,eosinophils, K/uL; BA, basophils, K/uL; T.Bil, total bilirubin, mg/dL; BUN, blood urea nitrogen, mg/dL; Crea, creatinine, mg/dL; UA, uric acid, mg/dL; Pi,

phosphorus, mg/dL; Glo, globulin, g/dL; Lipa, lipase, U/L; Glu, glucose, mg/dL; ALT, alanine transaminase, U/L; GGT, gamma-glutamyl transferase, U/L; LDH, lactate dehydrogenase, U/L; CHOL, colesterol, mg/dL; TG, triglycerides, mg/dL; Amyl, amylase, U/L; Cl-, chloride, mmol/L. (d) Tracking of the weights of animals during in vivo experiments. Although there was a sudden postoperative weight loss, as anticipated for major surgery, subsequent gradual weight gain with age suggested that the animals were healthy.

Supplementary Table 1. Summary of representative biodegradable polymers and hydrogels with their mechanical/degradation properties.

	Materials	T_g [°C]	T_m [°C]	Young's modulus [*E*, MPa]	Elongation at break [ε_b, %]	Degradation rate [weight loss in time]	Shelf life [in ambient conditions]	Issues	Ref
Biodegradable polymers	PLCL (50/50)	-7 - -3	-	2.5 - 7.5	900 - 1600	20 % in 30 weeks	> 6 month		Our works
	Silk	-	-	10000	20	35 % in 3 weeks	-	High modulus<br>Poor stretchability	[5]
	PLA	55	183	3000 - 3500	5 - 30	several years	5 years	High modulus<br>Poor stretchability	[6,7]
	PCL	-60	60-62	297 - 363	700-1000	several years	5 years	High modulus<br>Poor elasticity	[6,8,9]
	PGA	35-45	220	7000 - 8400	30	6 - 12 month	5 years	High modulus<br>Poor stretchability	[8]
	PLGA (50/50)	40-60	153	2000	3 - 10	1- 2 weeks	4 weeks in dry condition	High modulus<br>Poor stretchability<br>Rapid degradation	[7,10]
	PBS	5	163	700	6	10 % in 14 weeks	-	High modulus<br>Poor stretchability	[11,12]
	PHB	5	180	3500	5-8	60 % in 24 weeks	-	High modulus<br>Poor stretchability	[13]
	POC	0.2	-	0.92 - 16.4	260	50 % in 25 weeks	-	Low stretchability	[14]
	b-DCPU	-35 - -30	-	0.5 - 3.8	230	20 % in 10 weeks	-	Low stretchability	[15]
	PGS	-51.24, -18.50	5.23, 37.62	0.5	330	17 % in 60 days	-	Low stretchability	[16]
	PSeD-U	-20 - -2	-	0.54 - 0.74	136 - 509	60% in 8 hrs	-	Rapid degradation	[17]
Hydrogels	PGSA	-	-	0.143 - 0.592	20 - 130	90 % in 8 weeks	12 months	Low stretchability	[18]
	Gelatin biogel	-	-	0.03 - 0.3	320	60 % in 4 days	13 months	Low stretchability<br>Rapid degradation	[19]
	TRn11	-	-	2.4	300	on demand	-	Low stretchability<br>Poor storage stability	[20]
	GelMa	-	-	0.01 – few tens	150 - 290	10 days	6 months	Low stretchability<br>Rapid degradation<br>Poor storage stability	[21,22]
	HA-PVA	-	200-250	2.5	2900	N/A	-	Poor degradation<br>Poor storage stability	[23]
	BTIM	-	-	0.03	1200	20 days	-	Rapid degradation<br>Poor storage stability	[24]

Supplementary Table 2. Different molecular weights of PLCL elastomers synthesized with various monomer to initiator ratios.

	Molar ratio		Mn	Mw	PDI
	Monomers (CL:LA=5:5)	Initiator			
60k PLCL	5000	1	62112	88199	1.42
140k PLCL	10000	1	141922	208419	1.47
200k PLCL	20000	1	196331	320020	1.63

Supplementary References

1. Sabbatier, G. *et al.* Design, Degradation Mechanism and Long-Term Cytotoxicity of Poly (l-lactide) and Poly (Lactide-co-ϵ-Caprolactone) Terpolymer Film and Air-Spun Nanofiber Scaffold. *Macromol. Biosci.* **15**, 1392-1410 (2015).
2. Leja, K. & Lewandowicz, G. Polymer biodegradation and biodegradable polymers-a review. *Pol. J. Environ. Stud.* **19**, 255-266 (2010).
3. Liu, Y. *et al.* Soft and elastic hydrogel-based microelectronics for localized low-voltage neuromodulation. *Nat. Biomed.* **3**, 58 (2019).
4. Voigt, J-U., & Marta, C. 2-and 3-dimensional myocardial strain in cardiac health and disease. JACC: *Cardiovascular Imaging* **12**, 1849-1863 (2019).
5. Vepari, C. & Kaplan, D. L. Silk as a biomaterial. *Prog. Polym. Sci.* **32**, 991–1007 (2007).
6. Sarazin, P., Roy, X. & Favis, B. D. Controlled preparation and properties of porous poly (L-lactide) obtained from a co-continuous blend of two biodegradable polymers. *Biomaterials* **25**, 5965-5978 (2004).
7. Gentile, P. *et al.* An Overview of Poly(lactic-co-glycolic) Acid (PLGA)-Based Biomaterials for Bone Tissue Engineering. *Int. J. Mol. Sci.* **15**, 3640-3659 (2014).
8. Manavitehrani, I. *et al.* Biomedical Applications of Biodegradable Polyesters. *Polymers* **8**, 20 (2016).
9. Eshraghi, S. & Das, S. Mechanical and microstructural properties of polycaprolactone scaffolds with one-dimensional, two-dimensional, and three-dimensional orthogonally oriented porous architectures produced by selective laser sintering. *Acta Biomater.* **6**, 2467-2476 (2010).
10. Houchin, M. L., & E. M. Topp. Physical Properties of PLGA Films During Polymer Degradation. *J. Appl. Polym. Sci.* **114**, 2848-2854 (2009).

11. Lindström, A., Albertsson, AC. & Hakkarainen, M. Quantitative determination of degradation products an effective means to study early stages of degradation in linear and branched poly (butylene adipate) and poly (butylene succinate). *Polym. Degrad. Stab.* **83**, 487-493 (2004).

12. Liu, L. *et al.* Mechanical properties of poly (butylene succinate) (PBS) biocomposites reinforced with surface modified jute fibre. *Compos. - A: Appl. Sci. Manuf.* **40**, 669-674 (2009).

13. Volova, T. *et al.* Results of biomedical investigations of PHB and PHB/PHV fibers. *Biochem. Eng. J.* **16**, 125-133 (2003).

14. Yang, J., Webb, A. R. & Ameer, G. A. Novel citric acid-based biodegradable elastomers for tissue engineering. *Adv. Mater.* **16**, 511-516 (2004).

15. Choi, Y. S. *et al.* Stretchable, dynamic covalent polymers for soft, long-lived bioresorbable electronic stimulators designed to facilitate neuromuscular regeneration. *Nat. Commun.* **11**, 1–14 (2020).

16. Wang, Y. *et al.* A tough biodegradable elastomer. *Nat. Biotechnol.* **20**, 602-606 (2002).

17. Chen, S. *et al.* Mechanically and biologically skin-like elastomers for bio-integrated electronics. *Nat. Commun.* **11**, 1–8 (2020).

18. Held, M. *et al.* Soft electronic platforms combining elastomeric stretchability and biodegradability. *Adv. Sustain. Syst.* **6**, 2100035 (2022).

19. Baumgartner, M. *et al.* Resilient yet entirely degradable gelatin-based biogels for soft robots and electronics. *Nat. Mater.* **19**, 1102–1109 (2020).

20. Pena-Francesch, A., Jung, H., Demirel, M. C. & Sitti, M. Biosynthetic self-healing materials for soft machines. *Nat. Mater.* **19**, 1230–1235 (2020).

21. Van Den Bulcke, AI. *et al.* Structural and rheological properties of methacrylamide modified gelatin hydrogels. *Biomacromolecules* **1**, 31-38 (2000).

22. Kong, B. *et al.* Fiber reinforced GelMA hydrogel to induce the regeneration of corneal stroma. *Nat. Commun.* **11**, 1-12 (2020).

23. Hua, M. *et al.* Strong tough hydrogels via the synergy of freeze-casting and salting out. *Nature* **590**, 594-599 (2021).

24. Yang, Q. *et al.* Photocurable bioresorbable adhesives as functional interfaces between flexible bioelectronic devices and soft biological tissues. *Nat. Mater.* **20**, 1559-1570 (2021).
