# Supplementary material for: Ultra-stretchable and biodegradable elastomers for soft, transient electronics
Source: Nat Commun. 2023 Apr 20;14:2263. doi: 10.1038/s41467-023-38040-4 (PMC10119106; doi:10.1038/s41467-023-38040-4)
Supplement: Supplementary file 3 — Description to Additional Supplementary Information [file 41467_2023_38040_MOESM3_ESM.pdf]

Description of Additional Supplementary Files

Supplementary Movie 1. Deformable electronic display on PLCL

Supplementary Movie 2. PLCL-PP conductive elastomer

Supplementary Movie 3. A soft gripper

Supplementary Movie 4. Soft, transient electronic actuator (temp)

Supplementary Movie 5. Soft, transient electronic actuator (strain)

Supplementary Movie 6. Soft, transient, suture-free cardiac jacket
