# Supplementary material for: Ultra-stretchable and biodegradable elastomers for soft, transient electronics
Source: Nat Commun. 2023 Apr 20;14:2263. doi: 10.1038/s41467-023-38040-4 (PMC10119106; doi:10.1038/s41467-023-38040-4)
Supplement: Supplementary file 10 — Reporting Summary [file 41467_2023_38040_MOESM10_ESM.pdf]

nature portfolio

Corresponding author(s): Suk-Won Hwang

Last updated by author(s): Apr 9, 2023

Reporting Summary

Nature Portfolio wishes to improve the reproducibility of the work that we publish. This form provides structure for consistency and transparency in reporting. For further information on Nature Portfolio policies, see our Editorial Policies and the Editorial Policy Checklist.

Statistics

For all statistical analyses, confirm that the following items are present in the figure legend, table legend, main text, or Methods section.

n/a	Confirmed	
☐	☒	The exact sample size (*n*) for each experimental group/condition, given as a discrete number and unit of measurement
☐	☒	A statement on whether measurements were taken from distinct samples or whether the same sample was measured repeatedly
☒	☐	The statistical test(s) used AND whether they are one- or two-sided<br>*Only common tests should be described solely by name; describe more complex techniques in the Methods section.*
☒	☐	A description of all covariates tested
☐	☒	A description of any assumptions or corrections, such as tests of normality and adjustment for multiple comparisons
☐	☒	A full description of the statistical parameters including central tendency (e.g. means) or other basic estimates (e.g. regression coefficient) AND variation (e.g. standard deviation) or associated estimates of uncertainty (e.g. confidence intervals)
☒	☐	For null hypothesis testing, the test statistic (e.g. *F*, *t*, *r*) with confidence intervals, effect sizes, degrees of freedom and *P* value noted<br>*Give P values as exact values whenever suitable.*
☒	☐	For Bayesian analysis, information on the choice of priors and Markov chain Monte Carlo settings
☒	☐	For hierarchical and complex designs, identification of the appropriate level for tests and full reporting of outcomes
☒	☐	Estimates of effect sizes (e.g. Cohen's *d*, Pearson's *r*), indicating how they were calculated

Our web collection on statistics for biologists contains articles on many of the points above.

Software and code

Policy information about availability of computer code

Data collection	EMG and surface and epicardial ECGs were measured using a custom LabVIEW program that can be downloaded or free on Github:https://github.com/gwanjinko/biosignal.git.<br>PLCL/Mo-based voice and purse recordings, pressure sensing , and myocardial strain sensing were carried out using a 2600 series sensor program (IVsolution, Inc.)<br>Characterization of CMOS was conducted using a STAr sagittarius (STAr technologies, Inc. version 2016) program.<br>8×16 RGB LED matrix array was operated using a custom Aduino Genuino program that can be downloaded or free on Github:https://github.com/gwanjinko/LED-control.git.<br>Left ventricular ejection fractions were collected using a LabChart (ADinstruments, version 8).
Data analysis	Data analysis and plots were generated using Origin (version 2018 64 bit).<br>Mechanical simulation data was obtained using ABAQUS (ABAQUS 2017).

For manuscripts utilizing custom algorithms or software that are central to the research but not yet described in published literature, software must be made available to editors and reviewers. We strongly encourage code deposition in a community repository (e.g. GitHub). See the Nature Portfolio guidelines for submitting code & software for further information.

Data

Policy information about availability of data

All manuscripts must include a data availability statement. This statement should provide the following information, where applicable:

- Accession codes, unique identifiers, or web links for publicly available datasets
- A description of any restrictions on data availability
- For clinical datasets or third party data, please ensure that the statement adheres to our policy

All data supporting the findings and conclusions of this study are available within the paper and its Supplementary Information files. All other relevant data are available from the corresponding author upon request.

Human research participants

Policy information about studies involving human research participants and Sex and Gender in Research.

Reporting on sex and gender	N/A
Population characteristics	N/A
Recruitment	N/A
Ethics oversight	N/A

Note that full information on the approval of the study protocol must also be provided in the manuscript.

Field-specific reporting

Please select the one below that is the best fit for your research. If you are not sure, read the appropriate sections before making your selection.

☒ Life sciences ☐ Behavioural & social sciences ☐ Ecological, evolutionary & environmental sciences

For a reference copy of the document with all sections, see nature.com/documents/nr-reporting-summary-flat.pdf

Life sciences study design

All studies must disclose on these points even when the disclosure is negative.

Sample size	For in vivo demonstrations (Fig. 5d-h and Supplementary Fig. 29e, 30, 32, 33, and 34), a minimum number of n=3 biologically independent rats per group were utilized.<br>No statistical methods were used to pre-determine the sample size, since animal tests were intended to evaluate the stable in vivo operation of the fabricated devices.<br>Furthermore, the data obtained from this sample size was nearly consistent and reproducible, so we concluded that the sample size was sufficient.
Data exclusions	No data points were excluded from analysis.
Replication	At weeks 0, 1, 2, 4, and 8 weeks of device implantation, ECG and strain detection and electrical stimulation were performed by devices implanted in 3 biologically independent animals per groups at 1-day intervals.<br>Experiments were performed similarly across groups of animals and relevant data was successfully replicated (reproduced) to reliably support conclusions stated in the manuscript.
Randomization	All devices and animals were randomized into experimental groups. Groups were counterbalanced for group average body weight. Additional information is provided in the Online Methods section and in the Supplementary Note.
Blinding	All investigators were blinded to group allocation during data collection.<br>All assessments of device functionality (ECG, strain, electrical stimulation) and biocompatibility (histology sampling, ventricular ejection fraction measurement, etc.) were performed in a blinded manner.

Reporting for specific materials, systems and methods

We require information from authors about some types of materials, experimental systems and methods used in many studies. Here, indicate whether each material, system or method listed is relevant to your study. If you are not sure if a list item applies to your research, read the appropriate section before selecting a response.

Materials & experimental systems

n/a	Involved in the study
☒	☐ Antibodies
☒	☐ Eukaryotic cell lines
☒	☐ Palaeontology and archaeology
☐	☒ Animals and other organisms
☒	☐ Clinical data
☒	☐ Dual use research of concern

Methods

n/a	Involved in the study
☒	☐ ChIP-seq
☒	☐ Flow cytometry
☒	☐ MRI-based neuroimaging

Animals and other research organisms

Policy information about studies involving animals; ARRIVE guidelines recommended for reporting animal research, and Sex and Gender in Research

Laboratory animals	The study used 20 Sprague-Dawley rats (male, 6 weeks old, 180 ~ 200 g) that were purchased from Orientbio (South Korea). The animals were stabilized for 1 week, bred for more 6 weeks to gain a weight to ~550 g for unchanged heart size, and randomly divided into 5 groups: control and 1-, 2-, 4-, and 8-weeks.
Wild animals	The study did not involve wild animals.
Reporting on sex	The study utilized male animals. There was no need to consider sex, since animal tests were intended to evaluate the stable in vivo operation of the fabricated devices.
Field-collected samples	The study did not involve samples collected from the field.
Ethics oversight	All animal experiments were approved by the Institutional Animal Care and Use Committee (approval number: KOREA-2022-0022), Korea University, and followed the ethical principles for animal experimentation established by the institute.

Note that full information on the approval of the study protocol must also be provided in the manuscript.
